# Testing an Intervention to Improve Health Care Worker Well-Being During the COVID-19 Pandemic: A Cluster Randomized Clinical Trial

**DOI:** 10.1001/jamanetworkopen.2024.4192

**Published:** 2024-04-30

**Authors:** Lisa S. Meredith, Sangeeta Ahluwalia, Peggy G. Chen, Lu Dong, Carrie M. Farmer, Kathryn E. Bouskill, Sarah Dalton, Nabeel Qureshi, Tara Blagg, George Timmins, Lucy B. Schulson, Shreya S. Huilgol, Bing Han, Stephanie Williamson, Patricia Watson, Paula P. Schnurr, Monique Martineau, Katie Davis, Andrea Cassells, Jonathan N. Tobin, Courtney Gidengil

**Affiliations:** 1 RAND Corporation, Santa Monica, California; 2 RAND Corporation, Pittsburgh, Pennsylvania; 3 Section of General Internal Medicine, Chobanian & Avedisian School of Medicine, Boston University, Boston, Massachusetts; 4 RAND Corporation, Boston, Massachusetts; 5 Department of Research & Evaluation, Southern California Kaiser Permanente, Pasadena; 6 National Center for PTSD, White River Junction, Vermont; 7 Geisel School of Medicine at Dartmouth, Hanover, New Hampshire; 8 RAND Corporation, Arlington, Virginia; 9 Vizient Inc, Irving, Texas; 10 Clinical Directors Network, New York, New York; 11 The Rockefeller University Center for Clinical and Translational Science, New York, New York

## Abstract

**Importance:**

Stress First Aid is an evidence-informed peer-to-peer support intervention to mitigate the effect of the COVID-19 pandemic on the well-being of health care workers (HCWs).

**Objective:**

To evaluate the effectiveness of a tailored peer-to-peer support intervention compared with usual care to support HCWs’ well-being at hospitals and federally qualified health centers (FQHCs) during the COVID-19 pandemic.

**Design, Setting, and Participants:**

This cluster randomized clinical trial comprised 3 cohorts of HCWs who were enrolled from March 2021 through July 2022 at 28 hospitals and FQHCs in the US. Participating sites were matched as pairs by type, size, and COVID-19 burden and then randomized to the intervention arm or usual care arm (any programs already in place to support HCW well-being). The HCWs were surveyed before and after peer-to-peer support intervention implementation. Intention-to-treat (ITT) analysis was used to evaluate the intervention’s effect on outcomes, including general psychological distress and posttraumatic stress disorder (PTSD).

**Intervention:**

The peer-to-peer support intervention was delivered to HCWs by site champions who received training and subsequently trained the HCWs at their site. Recipients of the intervention were taught to respond to their own and their peers’ stress reactions.

**Main Outcomes and Measures:**

Primary outcomes were general psychological distress and PTSD. General psychological distress was measured with the Kessler 6 instrument, and PTSD was measured with the PTSD Checklist.

**Results:**

A total of 28 hospitals and FQHCs with 2077 HCWs participated. Both preintervention and postintervention surveys were completed by 2077 HCWs, for an overall response rate of 28% (41% at FQHCs and 26% at hospitals). A total of 862 individuals (696 females [80.7%]) were from sites that were randomly assigned to the intervention arm; the baseline mean (SD) psychological distress score was 5.86 (5.70) and the baseline mean (SD) PTSD score was 16.11 (16.07). A total of 1215 individuals (947 females [78.2%]) were from sites assigned to the usual care arm; the baseline mean (SD) psychological distress score was 5.98 (5.62) and the baseline mean (SD) PTSD score was 16.40 (16.43). Adherence to the intervention was 70% for FQHCs and 32% for hospitals. The ITT analyses revealed no overall treatment effect for psychological distress score (0.238 [95% CI, −0.310 to 0.785] points) or PTSD symptom score (0.189 [95% CI, −1.068 to 1.446] points). Post hoc analyses examined the heterogeneity of treatment effect by age group with consistent age effects observed across primary outcomes (psychological distress and PTSD). Among HCWs in FQHCs, there were significant and clinically meaningful treatment effects for HCWs 30 years or younger: a more than 4-point reduction for psychological distress (−4.552 [95% CI, −8.067 to −1.037]) and a nearly 7-point reduction for PTSD symptom scores (−6.771 [95% CI, −13.224 to −0.318]).

**Conclusions and Relevance:**

This trial found that this peer-to-peer support intervention did not improve well-being outcomes for HCWs overall but had a protective effect against general psychological distress and PTSD in HCWs aged 30 years or younger in FQHCs, which had higher intervention adherence. Incorporating this peer-to-peer support intervention into medical training, with ongoing support over time, may yield beneficial results in both standard care and during public health crises.

**Trial Registration:**

ClinicalTrials.gov Identifier: NCT04723576

## Key Points

**Question:**

Did Stress First Aid, a peer-to-peer support intervention, improve the well-being of health care workers (HCWs) compared with usual care during the COVID-19 pandemic?

**Findings:**

In this cluster randomized clinical trial of 28 hospitals and federally qualified health centers (FQHCs) with 2077 HCWs, intent-to-treat analyses revealed no overall treatment effect of the intervention. In post hoc analyses, HCWs aged 30 years or younger who received the intervention in FQHCs had significantly less psychological distress and posttraumatic stress disorder.

**Meaning:**

Findings of this study indicate that incorporating this peer-to-peer support intervention into medical training, with ongoing support over time, may yield beneficial results in both standard care and during public health crises.

## Teaser

This cluster randomized clinical trial evaluates the implementation of a peer-to-peer support program to reduce psychological distress and posttraumatic stress disorder among patient-facing health care workers at US hospitals and federally qualified health centers.

## Introduction

Health care workers (HCWs) faced unprecedented stressors during the COVID-19 pandemic.^1,2,3^ They frequently worked long hours in isolation while wearing personal protective equipment that was, at times, inadequate. They also had to maintain constant vigilance against the spread of infectious disease, not only to themselves, but also to their patients and families. Many HCWs also faced competing medical and personal demands,^4,5^ often while coping with exhaustion, fear, grief, and the moral distress of difficult triage decisions.^6,7,8,9^ Conditions improved with the availability of effective vaccines and evidence-based treatments for COVID-19. However, HCWs continued to experience high levels of burnout and traumatic stress,^10^ which may have been even higher than prepandemic levels,^11^ due to staffing shortages,^12^ politicization of masking and treatments, vaccine misinformation, workplace violence,^13^ and surges due to subsequent COVID-19 variants.^14,15^ Meanwhile, systemic factors of burnout, such as increasing clinical and administrative workloads as well as lack of support from the public and health system leadership, continue to threaten the well-being of HCWs.^16^ Not surprisingly, HCWs experience high rates of mental health problems,^17^ including generalized anxiety disorder.^18^

Stress First Aid is a promising evidence-informed intervention for mitigating the psychosocial effect of COVID-19 on HCWs.^19,20,21,22,23,24^ Initially developed for the US Navy and US Marine Corps,^19,25,26^ this intervention is a framework of actions for peer support delivered by individuals without mental health training to mediate and mitigate the effect of atypically stressful circumstances, but it was not designed to prevent any particular mental health disorder. Additionally, this peer-to-peer support intervention has been adapted to integrate seamlessly into the health care environment for HCW self-care and to help HCWs support patients and families. This intervention can be rapidly deployed, is actionable, and is generalizable to different types of HCWs in a variety of roles during the COVID-19 pandemic. In this cluster randomized clinical trial (RCT), we aimed to evaluate the effectiveness of a tailored peer-to-peer support intervention compared with usual care to support HCWs’ well-being at hospitals and federally qualified health centers (FQHCs) during the pandemic.

## Methods

### Trial Design, Oversight, and Population

This cluster RCT was conducted at hospitals and FQHCs throughout the US.^27^ The trial comprised 3 sequential cohorts, starting 1 year into the pandemic in March 2021 through the latter stage of the pandemic in July 2022, to allow for rapid integration of lessons learned during the study. The RAND Institutional Review Board (IRB) and IRBs at any of the 28 participating sites that could not cede to RAND’s IRB approved the trial protocol (Supplement 1). Trial oversight was provided by a study-specific Data Safety and Monitoring Board, which reviewed study progress and accumulating data. The survey contained consent language, and completion of the survey implied consent. In accordance with the Common Rule, consent for participation in the intervention was not required because the it was delivered by hospital and FQHC staff to HCWs within the sites using a train-the-trainer approach and not directly by the research team. We followed the Consolidated Standards of Reporting Trials (CONSORT) reporting guideline.

**Figure 1.**
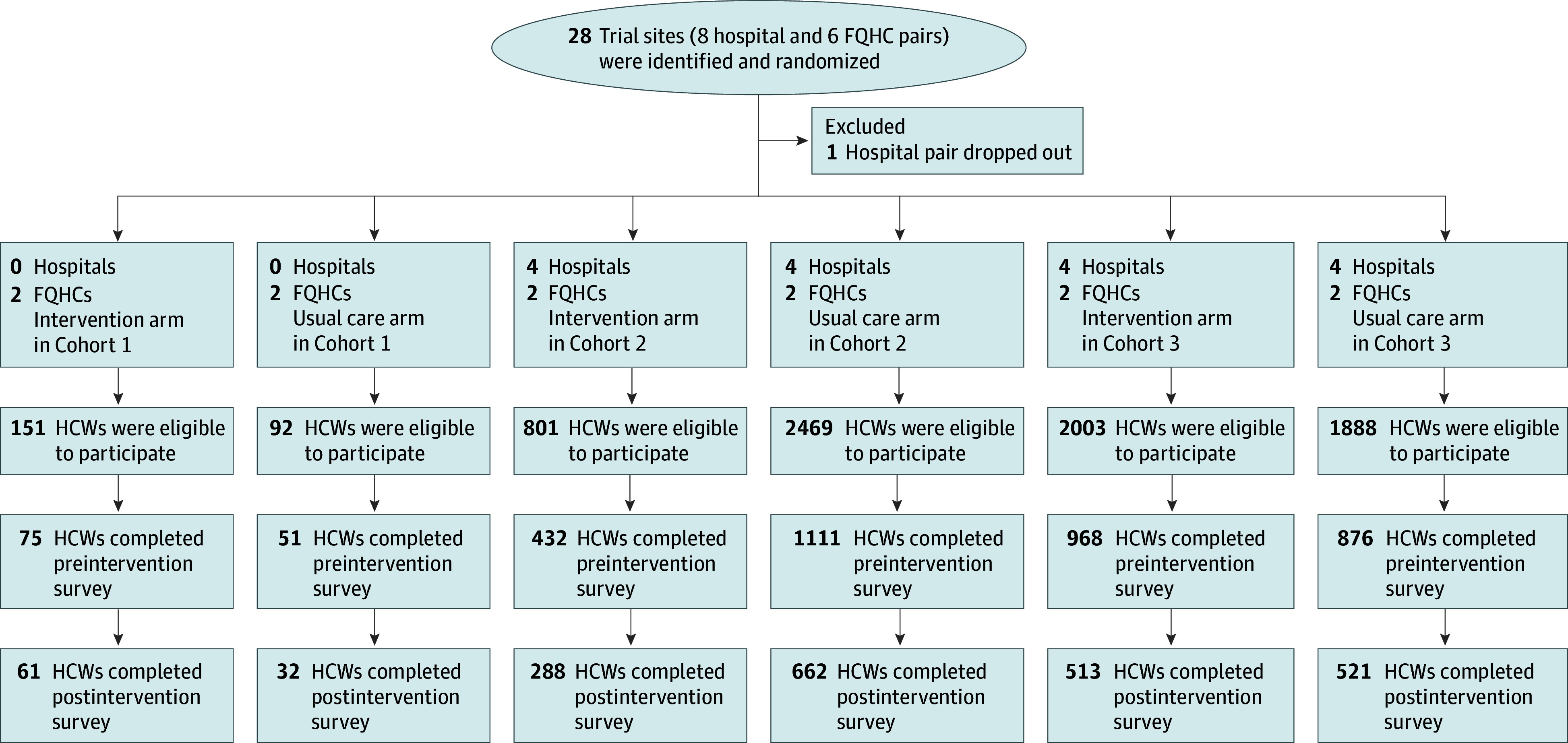
Trial Recruitment and Participation Flowchart FQHC, Federally Qualified Health Center; HCW, health care worker.

Sites were recruited as pairs, matched on the basis of size (number of beds for hospitals and number of patients for FQHCs), type (teaching vs non-teaching), and COVID-19 burden (using zip code as a proxy). Sites could be within the same health system or from different health systems. Each site within a pair was randomized to the intervention arm or usual care arm (Figure 1).

Within hospitals, specific clinical teams or units (eg, intensive care unit, emergency department, or general floors) were identified with input from site leaders and champions about who should participate. Eligible participants were HCWs within these teams or units and included physicians, nurse practitioners, physician assistants, nurses, and respiratory therapists. Hospital sites in the usual care arm then included the same teams or units as their matched intervention sites. Within FQHCs, all patient-facing staff participated, including both HCWs and support staff (eg, front desk staff). Staff who were not directly patient-facing were excluded from the trial and all data collection.

### Trial Intervention

The peer-to-peer support intervention is framed around 5 evidence-informed elements that foster recovery from trauma, loss, and other types of adversity, promoting a sense of safety, calm, self-efficacy, community efficacy, and hope.^28^ It operationalizes these 5 elements into flexible actions that can be easily remembered and enacted by those working in a high-stress and ongoing-stress environment. Recipients of the peer-to-peer support intervention are taught to respond to their own and their peers’ stress reactions using 7 core actions: check, coordinate, cover, calm, connect, competence, and confidence.^29^ Additional details about the intervention are provided in the Study Plan in Supplement 1.

We used a train-the-trainer approach to deploy the peer-to-peer support intervention. The approach involved identifying at least 1 champion per every 50 HCWs to receive the intervention training. Site champions reviewed 4 hours of training videos and then participated in a 2-hour virtual, live peer-to-peer support training delivered by the developer of the adapted intervention (P.W.). Champions received a handbook describing the implementation and sustainment strategies for providing peer-to-peer support intervention training locally to their teams. After training, the champions began training their peers at their own organizations. Sites that were randomized to usual care continued providing any existing programs to support their HCWs during and beyond the COVID-19 pandemic.

### Primary and Secondary Outcomes

All HCWs received a web-based outcomes survey before and after intervention implementation. Surveys remained in the field for a mean (range) of 41 (29-53) days, with longer fielding periods around the holidays and a site-requested delay for some sites due to the Omicron variant surge (eTable 1 in Supplement 2).

The primary outcomes were general psychological distress and posttraumatic stress disorder (PTSD). We measured general psychological distress with the Kessler 6,^30^ which is both a symptom measure, with a score range of 0 to 24 (with the highest scores indicating higher distress), and a binary measure of serious psychological distress, with the standard cutoff of 13 (indicating distress). We measured PTSD using the PTSD Checklist (PCL-5),^31^ which scores PTSD in 3 ways: by symptom severity (possible range: 0-80, with the highest scores indicating greater symptom severity); as a binary measure of probable PTSD, with the standard cutoff of 33 (indicating that the PTSD criteria were met); and as a binary measure to indicate that the clinical criteria of the *DSM-5* (*Diagnostic and Statistical Manual of Mental Disorders* [Fifth Edition])^31^ were met.

Secondary outcomes included sleep-related impairment,^32^ workplace stress,^33,34^ burnout (using a single item with 5 response options ranging from 1 [I enjoy my work. I have no symptoms of burnout.] to 5 [I feel completely burned out.]),^33,35^ resilience, and moral distress.^36^ We collected information about experiences with COVID-19.^37^ We also collected HCWs’ self-reported demographic (age group, gender, and race and ethnicity [Black; Hispanic or Latino/Latina; White; and other, which was not defined, but with an option to write in]) and professional (role and years employed at site and in profession) characteristics. We included demographic and professional characteristics to describe the sample and to serve as control variables so that multivariable models accounted for variation in HCW characteristics.

### Sample Size and Power Calculation

This study planned for a sample size of 1552 to 2520 completed surveys in both waves and from 44 to 54 sites. Under the setting of 2-sided type I error rate of less than 0.05 and power greater than 0.8 and assuming an intraclass correlation of 0.01 within each site, the detectable effect sizes ranged between 0.14 and 0.31 times SD for overall and subgroup analyses (by type of setting: hospitals and FQHCs), with 2 equal-sized subgroups. Post hoc power was based on the actual sample size (n = 2077 completed surveys). Under the same setting in previous power calculations, the detectable effect sizes were 0.17 SD for overall analysis, 0.20 SD for hospitals, and 0.32 SD for FQHCs. These standardized effect sizes corresponded to 0.94, 1.10, and 1.76 points, respectively, for the PCL-5 score (SD, approximately 5.5). We also conducted stratified analyses by age groups within each setting. The post hoc power calculation was based on one-third of the actual sample size in each setting. The minimum detectable effect sizes were 0.28 SD for hospitals and 0.50 SD for FQHCs. These standardized effect sizes translated to 1.54 and 2.75 points, respectively, for the PCL-5 score.

### Statistical Analysis

Our primary analyses were based on participants with complete data. We first excluded the few incomplete records with item-level missing data from our analyses. We handled partial responders, in which participants did not complete the postintervention survey, through sensitivity analysis using the data-reweighting technique to adjust for statistical estimates. However, in all sensitivity check models, the estimates were highly similar to the results from complete-case models. Thus, for simplicity we used the complete-case analysis as the main results.

Our primary approach was a preplanned, intent-to-treat (ITT), and difference-in-differences (DID) method, regardless of actual exposure levels to treatment. DID was robust to unobserved differences at the baseline and temporal patterns unrelated to treatment. The DID analysis was implemented through mixed-effect longitudinal models with 2 nested random effects for individual-level and site-level correlations. We estimated the treatment effect by the interaction between treatment status and follow-up wave indicator. This DID effect is on a linear scale for continuous variables and is a log odds ratio for binary variables. We controlled for all of the baseline covariates in Table 1 in all models. Due to the inherent differences in setting, we conducted stratified DID analyses for FQHCs and hospitals separately. The 2 subgroup analyses were completely nonoverlapping and yielded statistically independent estimates and inference results. The overall treatment effect was estimated as a weighted mean of the 2 subgroup effect estimates, where weights were the sample size in each subgroup analysis. 

**Table 1.** Baseline Characteristics of Health Care Workers (HCWs) by Study Condition for Federally Qualified Health Centers (FQHCs) and Hospitals

Characteristic	HCWs at FQHCs, No. (%)			HCWs at hospitals, No. (%)		
	Full sample (n = 428)	Intervention (n = 245)	Usual care (n = 183)	Full sample (n = 1649)	Intervention (n = 617)	Usual care (n = 1032)
Demographic						
Age group, y						
≤30	101 (23.7)	72 (29.4)	29 (15.9)	397 (24.1)	151 (24.5)	246 (23.9)
31-50	236 (55.3)	130 (53.1)	106 (58.2)	946 (57.5)	356 (57.8)	590 (57.3)
≥51	90 (21.2)	43 (17.6)	47 (25.8)	302 (18.4)	109 (17.7)	193 (18.8)
Gender^a^						
Female	356 (83.4)	199 (81.2)	157 (86.3)	1287 (78.2)	497 (80.5)	790 (76.8)
Male	63 (14.8)	39 (15.9)	24 (13.2)	355 (21.6)	120 (19.4)	235 (22.8)
Race and ethnicity^b^						
Black	96 (22.4)	54 (22.0)	42 (23.0)	181 (11.0)	78 (12.6)	103 (10.0)
Hispanic or Latino/Latina	163 (38.1)	106 (43.3)	57 (31.1)	253 (15.3)	51 (8.3)	202 (19.6)
White	132 (30.8)	68 (27.8)	64 (35.0)	813 (49.3)	397 (64.3)	416 (40.3)
Other^c^	37 (8.6)	17 (6.9)	20 (10.9)	402 (24.4)	91 (14.7)	311 (30.1)
Professional						
Role						
Clinician^d^	90 (21.0)	31 (12.7)	59 (32.2)	206 (12.5)	46 (7.5)	160 (15.5)
Nurse	26 (6.1)	18 (7.3)	8 (4.4)	819 (49.7)	287 (46.5)	532 (51.6)
Assistant or technician	154 (36.0)	111 (45.3)	43 (23.5)	431 (26.1)	189 (30.6)	242 (23.4)
Administrative or other^e^	158 (36.9)	85 (34.7)	73 (39.9)	193 (11.7)	95 (15.4)	98 (9.5)
≤5 y Employed at the site	308 (72.3)	178 (73.0)	130 (71.4)	923 (56.0)	357 (57.9)	566 (55.0)
≤5 y In the profession	185 (43.3)	118 (48.4)	67 (36.6)	582 (35.4)	230 (37.3)	352 (34.2)

^a^ The sum of percentages in this category may not be 100 because data for cell sizes less than 5 were suppressed.

^b^ Race and ethnicity were self-reported in the surveys.

^c^ Other was not defined, but with an option to write in.

^d^ Clinician included physicians, nurse practitioners, and physician assistants.

^e^ Other included patient support specialists, behavioral health specialists, substance use counselors, pharmacists, educators, students, and speech therapists.

Additionally, we conducted data-driven subgroup analyses by age levels. During the preplanned analyses, we noticed strong correlations between age and primary outcomes. As an exploratory analysis, we examined whether age might moderate the treatment effects.

Two-sided *P* < .05 indicated statistical significance. Data analysis was performed using SAS, version 9.4 (SAS Institute, Inc).

## Results

### Trial Sites and Participants

Twenty-eight sites (8 hospital pairs, and 6 FQHC pairs) throughout the US were randomized to peer-to-peer support intervention or usual care. A total of 2077 HCWs from these facilities (428 for FQHCs, and 1649 for hospitals) participated, completing both the preintervention and postintervention surveys, for a 28% overall response rate (41% for FQHCs, and 26% for hospitals) (Figure 1). The response rate was 48% for the preintervention survey and 62% for the postintervention survey. Adherence to the intervention (the percentage of HCWs who reported participating in the peer-to-peer support intervention on the postintervention survey) was 70% for FQHCs and 32% for hospitals.

Sample baseline characteristics of HCWs from FQHCs and hospitals are shown overall and by study condition in Table 1. A total of 862 individuals (696 females [80.7%] and 159 males [18.4%]) were from sites randomly assigned to the intervention arm; the baseline mean (SD) psychological distress score was 5.86 (5.70) and the baseline mean (SD) PTSD score was 16.11 (16.07). A total of 1215 individuals (947 females [78.2%] and 259 males [21.4%]) were from sites assigned to the usual care arm; the baseline mean (SD) psychological distress score was 5.98 (5.62) and the baseline mean (SD) PTSD score was 16.40 (16.43). With site-level randomization, characteristics were not balanced across arms at the individual HCW level. The FQHC sample comprised 356 females (83.4%) and 63 males (14.8%). The hospital sample comprised 1287 females (78.2%), 355 males (21.6%). In this group, HCWs were younger and more likely to have been in the profession for at least 5 years compared with their counterparts at hospitals. Among HCWs at FQHCs, 96 (22.4%) self-identified as Black, 163 (38.1%) as Hispanic or Latino/Latina, 132 (30.8%) as White, and 37 (8.6%) as other race and ethnicity. Among HCWs at hospitals, 181 (11.0%) self-identified as Black, 253 (15.3%) as Hispanic or Latino/Latina, 813 (49.3%) as White, and 402 (24.4%) as other race and ethnicity. Across all sites, more HCWs in the intervention arm were administrators and assistants or technicians rather than physicians, nurse practitioners, physician assistants, and nurses.

### Treatment Effect

eTables 2 and 3 in Supplement 2 show unadjusted preintervention and postintervention outcomes by treatment arm for HCWs at FQHCs and hospitals. Table 2 shows the adjusted treatment effects from the planned ITT analysis, where an estimate with a positive value indicates that the outcome was higher for the intervention than for usual care. Because sample weights made no difference in the outcomes, the data were adjusted for covariates but were unweighted for nonresponse. The ITT analyses revealed no overall treatment effect for psychological distress score (0.238 [95% CI, −0.310 to 0.785] points) or PTSD symptom score (0.189 [95% CI, −1.068 to 1.446] points). We found no treatment effect for any of the outcomes or when analyzing the data separately by site type, although the effects were more pronounced for FQHCs than for hospitals, with 1 exception for the hospital sample: HCWs in the intervention arm had a larger increase in meeting clinical criteria for PTSD over the study period than did those in the UC arm (unadjusted increase from 16.6% to 18.6% in the intervention arm; unadjusted decrease from 18.7% to 16.1% in the usual care arm; adjusted DID estimate, 0.323 [95% CI, 0.030 to 0.616]).

**Table 2.** Intention-to-Treat Results for the Full Sample and by Facility Type^a^

Outcome measure	FQHCs (n = 428)		Hospitals (n = 1649)		Full sample (n = 2077)	
	Estimate (95% CI)	*P* value	Estimate (95% CI)	*P* value	Estimate (95% CI)	*P* value
Primary outcomes						
Psychological distress score (range: 0-24)^b^	−0.490 (−1.946 to 0.965)	.51	0.426 (−0.152 to 1.003)	.15	0.238 (−0.310 to 0.785)	.39
Serious psychological distress (cutoff: ≥13)^b^	−0.010 (−0.648 to 0.627)	.98	0.245 (−0.142 to 0.631)	.21	0.192 (−0.141 to 0.526)	.26
PTSD symptom score (range: 0-80)^b^	−0.897 (−3.416 to 1.623)	.48	0.468 (−0.975 to 1.912)	.52	0.189 (−1.068 to 1.446)	.77
PTSD provisional diagnosis (cutoff: ≥34)^b^	−0.183 (−0.875 to 0.508)	.60	0.255 (−0.041 to 0.550)	.09	0.165 (−0.109 to 0.439)	.24
PTSD (met *DSM-5* clinical criteria)	0.006 (−0.641 to 0.654)	.98	0.323 (0.030 to 0.616)	.03	0.258 (−0.010 to 0.526)	.06
Secondary outcomes						
Sleep-related impairment score (range: 4-20)^c^	−0.267 (−1.104 to 0.570)	.53	−0.223 (−0.658 to 0.212)	.31	−0.232 (−0.618 to 0.153)	.24
Workplace stress score (range: 4-20)^c^	0.032 (−0.428 to 0.493)	.89	0.160 (−0.089 to 0.408)	.21	0.134 (−0.085 to 0.352)	.23
Burnout, %	0.052 (−0.371 to 0.475)	.81	0.010 (−0.201 to 0.220)	.93	0.018 (−0.170 to 0.207)	.85
Resilience score (range: 0-8)^c^	−0.188 (−0.510 to 0.133)	.25	−0.015 (−0.154 to 0.124)	.83	−0.051 (−0.179 to 0.078)	.44
Moral distress score (range: 0-10)^c^	0.256 (−0.320 to 0.833)	.38	0.223 (−0.061 to 0.507)	.12	0.230 (−0.025 to 0.484)	.08

Abbreviations: *DSM-5, Diagnostic and Statistical Manual of Mental Disorders* (Fifth Edition); FQHC, Federally Qualified Health Center; PTSD, posttraumatic stress disorder.

^a^ Estimate refers to the estimated coefficient for the interaction term between treatment status and time. This difference-in-differences effect is on a linear scale for continuous variables and is a log odds ratio for binary variables. Models were adjusted for health care workers’ demographic (age group, gender, and race and ethnicity) and professional (role and years employed at site) characteristics in Table 1.

^b^ Psychological distress score range: 0-24, with the highest score indicating higher distress. Serious distress score cutoff (13) indicates distress. PTSD symptom score range: 0-80, with the highest score indicating greater symptom severity. PTSD provisional diagnosis cutoff (34) indicates that the PTSD criteria were met.

^c^ Sleep-related impairment score range: 4-20, with the highest score indicating more sleep impairment. Workplace stress score range: 4-20, with the highest score indicating more workplace stress. Resilience score range: 0-8, with the highest score indicating more resilience. Moral distress score range: 0-10, with the highest score indicating more moral distress.

**Figure 2.**
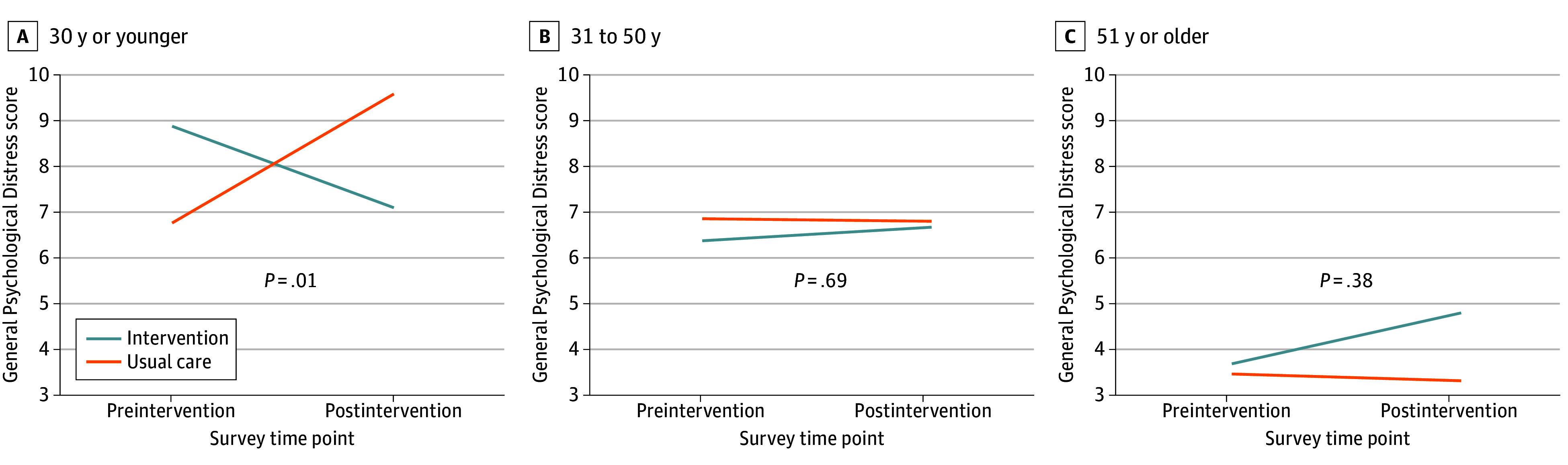
General Psychological Distress by Age in Federally Qualified Health Centers

**Figure 3.**
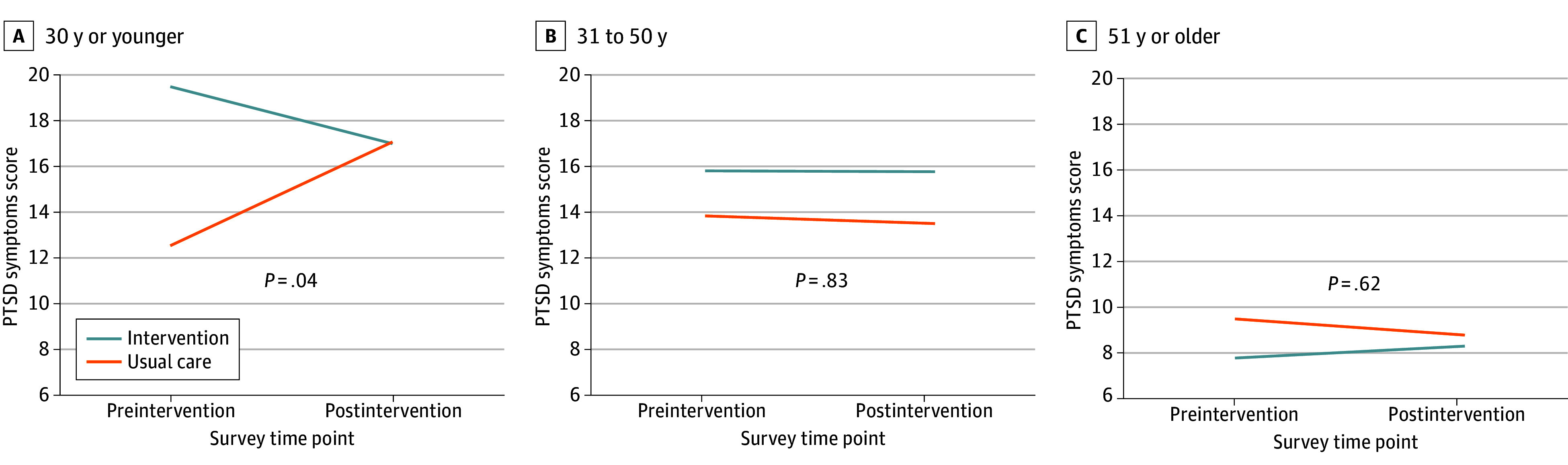
Posttraumatic Stress Disorder (PTSD) by Age in Federally Qualified Health Centers

We observed robust age effects across outcomes in the ITT analyses; post hoc analyses showed a consistent treatment effect by age group in FQHCs for HCWs aged 30 years or younger across the primary outcomes of general psychological distress and PTSD (eTable 4 in Supplement 2). The treatment effect was a 4.552-point reduction (95% CI, −8.067 to −1.037) on the 0 to 24 psychological distress score and a 6.771-point reduction (95% CI, −13.224 to −0.318) on the 0 to 80 PTSD symptom score, which were clinically meaningful effect sizes (Figure 2 and Figure 3).

## Discussion

To our knowledge, this RCT was the first to evaluate this peer-to-peer support intervention in HCWs with robust quantitative analyses. The ITT analyses revealed no overall treatment effect; however, in the FQHC sample, we found a significant treatment effect for younger HCWs (aged ≤30 years) that was consistent across the psychological distress and PTSD outcomes, but not for older HCWs. This finding is promising, particularly since skills gained through the peer-to-peer support intervention may have a long-term and lasting effect throughout the careers of younger HCWs.

This RCT was conducted during the COVID-19 pandemic, a unique period with inherent instability given the constantly shifting professional risks and challenges faced by HCWs. Thus, it is difficult to compare these findings to those from studies conducted during nonpandemic times or in settings where a peer-to-peer support intervention is implemented over a longer period and in a more flexible way. Because there is no other study of this peer-to-peer support intervention implemented and evaluated rigorously through an RCT during a pandemic, the finding that the intervention did not have a protective effect is somewhat hard to interpret, particularly given both variable adherence to the intervention and the low response rates. However, it does suggest that implementing a peer-to-peer support intervention and training HCWs during a pandemic and within the RCT constraints are unlikely to have a generally protective effect on HCWs’ well-being. Health care leaders who wish to pilot interventions such as the one described herein will need to tailor it to their own context, provide as much support as possible to maximize success, and consider incorporating it into training provided early on in HCWs’ careers.

Whether to provide training at all to improve well-being is another important question. This peer-to-peer support intervention is intended to support HCWs by creating a shared longitudinal framework for communication about stressors and challenges. On its own, the intervention cannot heal or prevent burnout in the absence of the systemic change needed to fundamentally improve the environment in which HCWs function.^38^ While this peer-to-peer support intervention should be helpful based on prior studies in non-HCW populations,^19,25,26^ brief training sessions and heightened awareness of stress without longer-term, purposeful support could paradoxically increase stress in the short-term,^39,40,41^ especially if systemic challenges either are not addressed or are ever-changing. Brief trainings during a lunch hour, for example, without systemic commitment to ongoing support, may worsen HCW stress, especially if the training identifies problems but is not seen as helpful in resolving those problems. Although the trial produced no direct quantitative evidence, its qualitative findings highlight the importance of leaders directly and practically supporting training, by providing protected time for trainings and meaningfully integrating commitment to the peer-to-peer support intervention.^42^ Commitment to providing training means that decision-makers will need to consider carefully how to truly shift culture toward genuine investment in HCWs’ well-being.

We recommend that future evaluations consider longitudinal assessment of ongoing intervention actions throughout the workplace as well as expansion of this peer-to-peer support intervention to the entire hospital rather than simply clinical units or teams. Setting a supportive and collaborative tone from the top down is important, in addition to having buy-in from the bottom up. Fielding the peer-to-peer support intervention to entire FQHCs may have partly contributed to its greater success in this setting compared with hospitals. Additionally, we recommend broadening the assessment to include changes in stigma in the work culture, coping self-efficacy, perceived organizational and social supports, well-being, and awareness and use of resources.

The age effect finding warrants further study, particularly within the context of COVID-19 and other pandemics. A recent systematic review found an inconsistent age effect across 53 studies (all conducted prior to 2020), although younger HCWs may be more likely to report burnout.^43^ One study that surveyed HCWs before and during the pandemic found that burnout and distress were more prevalent among HCWs with less experience.^44^ Given that we found a significant effect in younger HCWs in FQHCs, it could be helpful to focus future studies of this peer-to-peer support intervention on trainees (eg, residents and nursing trainees) to capture HCWs at a critical point in their early professional development, as the intervention was originally implemented in its first military setting to create culture change within a large system. Similarly, since it was challenging to implement the intervention in the middle of a pandemic, including it in prevention training or employee assistance programs may yield beneficial results in standard care and during public health crises..

### Limitations

This trial had several limitations. First, the 3 cohorts were less distinct than planned and many of the sites within different cohorts had overlapping timelines (eTable 1 in Supplement 2) because we strived to allow timeline flexibility when needed. For example, due to the Omicron variant wave in January 2022, we allowed hospitals whose efforts were well under way to pause implementation of the peer-to-peer support intervention and then resume with a targeted refresher to HCWs rather than to drop out of the trial, given enormous clinical demands. This issue affected both sites in a pair equally: the site assigned to usual care simply fielded their survey later (at the same time as the intervention site).

Second, the dose of the intervention may have been insufficient. Health care workers received a 1-hour in-person training, which was intended to be followed by 4 hours of booster time (four 1-hour booster sessions that could be operationalized as eight 30-minute sessions or even multiple shorter sessions) to ensure that the intervention was feasible in busy health care settings. This dose also aligned with other studies of this peer-to-peer support intervention.^7^ However, it is possible that no effect was observed because implementation was not sufficiently intensive. Typically, this peer-to-peer support intervention is offered as a 2- to 3-day training of trainers with follow-up consultations and guidance.

Third, the follow-up period may have been too short to capture the effect of the intervention on the chosen outcomes. This timing should not be an issue with a well-matched pair of sites, but appreciating the true effect of the intervention would still be difficult. Given that history is often the greatest threat to internal validity, comparing the results of implementation and assessment may have been confounded by the different experiences of multiple COVID-19 variants and waves.

Fourth, while the overall rate of response was low (only 28%) when considering the completion of both surveys, the response rates were high for preintervention (48%) and postintervention (62%) time points, especially considering the pandemic challenges. These rates may raise the possibility of selective response if HCWs who experienced benefit or whose well-being improved over time were less likely to complete follow-up surveys.

## Conclusions

The well-being of HCWs will remain a critical topic for years to come. The effects of the COVID-19 pandemic continue to reverberate throughout health care communities, aggravating the already strained health care system. US Surgeon General Dr Vivek Murthy identified workplace well-being as a top priority, specifically flagging HCW burnout.^45^ The Quadruple Aim posits that optimal “care of the patient requires care of the provider.”^46^ Protecting the well-being of HCWs as they face these myriad challenges while providing the best possible patient care has never been more important. In this trial, the peer-to-peer support intervention did not improve well-being outcomes for HCWs overall, but it was protective against general psychological distress and PTSD in HCWs 30 years or younger in FQHCs. Incorporating the intervention into medical training, with ongoing support over time, may yield beneficial results in both standard care and during public health crises.

## Notes

Supplement 1.
Trial Protocol

Supplement 2. **eTable 1.** Survey Fielding Dates by Cohort **eTable 2.** Unadjusted Outcomes Pre- and Post-Intervention by Treatment Status for HCWs in FQHCs **eTable 3.** Unadjusted Outcomes Pre- and Post-Intervention by Treatment Status for HCWs in Hospitals **eTable 4.** Intention-to-Treat Results by Age Group for the Full Sample and by Facility Type

Supplement 3.
Data Sharing Statement
