# Supplementary material for: Testing an Intervention to Improve Health Care Worker Well-Being During the COVID-19 Pandemic: A Cluster Randomized Clinical Trial
Source: JAMA Netw Open. 2024 Apr 30;7(4):e244192. doi: 10.1001/jamanetworkopen.2024.4192 (PMC11061774; doi:10.1001/jamanetworkopen.2024.4192)
Supplement: Supplement 1. — Trial Protocol [file jamanetwopen-e244192-s001.pdf]

COVER HCW
COVID-19 PROTECTION TO ENSURE RESILIENT HEALTH CARE WORKERS

Protecting the Mental and Physical Well-Being of Frontline Health Care Workers during COVID-19: Study Plan

Research Objectives

The goal of this project is to support the mental and physical well-being of U.S. health care workers (HCWs) during the COVID-19 pandemic to ensure high-quality care for patients, by establishing the effectiveness of a tailored Stress First Aid (SFA) intervention, compared to usual care (UC). The end result will be an SFA toolkit tailored for HCWs that can be implemented and scalable across multiple settings. Our specific aims are to:

(1) test the comparative effectiveness of SFA versus UC on mental and physical well-being (quantitative);
(2) understand and document any UC activities to support HCW well-being prior to implementing SFA across sites (qualitative); and
(3) assess the experiences of HCWs and sites with SFA (acceptability, likelihood of uptake, lessons learned) and impact on HCW well-being (qualitative).

Overview of Study Design

Our study uses a ***mixed methods approach*** that includes a <u>quantitative component</u> *(Aim 1)* designed as a cluster randomized controlled trial (cRCT) and a qualitative component *(Aims 2 & 3)* designed as a complementary descriptive study. The cRCT will be comprised of three cohorts of matched pairs representing approximately 40 diverse sites (12-15 pairs of hospitals hospitals and 5-7 pairs of clinics/practices) to determine whether SFA for frontline HCWs improves mental and physical well-being compared to Usual Care (UC). Sequential roll-out of the intervention to three cohorts will allow us to quickly incorporate lessons learned and stakeholder feedback from each iteration into subsequent trainings, and share actionable findings given the urgency due to the ongoing pandemic. Each recruited site will involve leadership (typically C-suite, e.g., executive level leaders to support and endorse the project) and HCWs. Intervention sites implementing SFA will also have champions who will train their peers.

The <u>quantitative component</u> will include the primary outcomes (PTSD and distress), based on established survey questions) as well as secondary outcomes (depression, anxiety, fatigue, sleep problems, and social roles/activities) to be captured by a HCW survey at both the SFA and UC sites before and after the intervention period.

The <u>qualitative component</u> will include in-depth interviews before and after the intervention with health care system leaders. These interviews will aid in understanding how particular contextual workplace environmental factors could intersect with the implementation and impact of the intervention, in turn ensuring that we can provide a more complete picture of the intervention's effectiveness and impact on HCWs and the organization writ large. We will conduct post-intervention interviews with HCWs at both SFA and UC sites to better understand how their well-being is affected by the workplace environment. In addition, we will interview champions to understand implementation of the SFA trainings locally at each site.

Study Population and Setting

We are partnering with Vizient Inc. and Clinical Directors Network (CDN) to recruit and engage sites. ***Vizient*** is a member-owned health care performance improvement organization serving more than half of all health care organizations across the country from which we will recruit 12-15 pairs of hospitals and 5-7 pairs of ambulatory practices. ***CDN*** is a practice-based research network from which we will recruit 5 pairs of FQHCs (we also call these practices for shorthand). ***Stanford Health Care (SHC)*** is a third partner that will provide both subject matter and

expertise and has committed to allowing us immediate and facilitated access to HCWs and leadership for pre-testing materials such as the survey, interview protocols, and intervention materials.

The training and evaluation will take place with approximately 22 pairs of sites (matched on size, type, geography, and COVID-19 burden): 12 pairs of hospitals and 5 pairs of Vizient-affiliated clinics, and 5 pairs of CDN-affiliated clinics. Leadership at recruited sites must agree to support the effort, including survey endorsement and encouragement and supporting time for all trainings.

Recruitment

Vizient will recruit members from their organization using targeted emails, supplemented by a live webinar (which will also be recorded and posted for members). CDN will reach out to practices as well using a listserv. Both organizations have access to a one-pager and slide set developed for this project which contain the same content but is tailored for each organization. Each site will be asked to identify leaders, champions, and ultimately HCWs:

- Site leaders will provide the study team with a birds-eye view of operations at the site, including the typical supports and resources the organization or system provides to maintain health care worker well-being. We expect that executive leadership (i.e., "C-suite" members) will likely be the most effective in this role, given their knowledge of operations. Possible site leaders include but are not limited to the Chief Quality Officer, Chief Nursing Officer, Chief Medical Officer, or Chief Operating Officer.
- Site champions will serve as SFA Trainers of health care worker teams in their hospital or practice, and be a resource to champions at other organizations via a virtual learning collaborative as trainings get under way. Champions will also serve as a conduit between their hospital or practice and the research team, helping to identify and address barriers and issues that arise during the SFA intervention

Recruitment started in late October 2022 and will continue until the third wave begins. Interested sites may be selected for any of the three waves, depending on the project needs to ensure balance across region and by other characteristics (e.g., academic status, bed size). While there are no formal strata, recruitment in later waves will be purposive to ensure some geographic and academic status diversity.

Sites recruited by CDN will be paid $10,000 for participation, which is consistent with prior project approaches (including PCORI-funded projects). This amount will be paid regardless of whether the practice is recruited to SFA or UC. Sites recruited by Vizient will not be paid an honorarium. Vizient and CDN also routinely arrange for CME/CNE credit rapidly, and will do so for champions and those HCWs they train in the SFA sites as an incentive to participate. HCWs at UC sites will not receive SFA or CME/CNE, unless they opt to adopt training after the intervention. HCWs will be paid $50 for interviews (if not receiving CME/CNE) and $25 per survey

Based on the above recruitment efforts, RAND and its partners will review the list of interested sites (hospitals and amulbatory practices, including FHQCs) from each organization. The list will be reviewed prior to each wave, acknowledging that additional targeted recruitment may be required in later waves to ensure appropriate matching of pairs (see below) and balance across region and academic status.

RCT Sampling Plan

Before randomizing, we will match sites into pairs for each of the three cohorts based on the following characteristics:

(1) Size (number of beds for hospitals & number of patients for FQHCs/clinics)
(2) Type (teaching vs. non-teaching)
(3) COVID-19 burden (using zip code or city as a proxy)

Each pair will then be assigned to either SFA or UC using a simple 1:1 randomization. We will administer a web-based survey to HCWs at all sites before and after the intervention. In addition, we will conduct a set of interviews with all site leaders (in SFA and UC sites) before and after the intervention period; and with champions (intervention sites only) and

a sub-sample of HCWs (intervention and control sites) after the intervention period. The figure below summarizes the study design and recruitment plan for the cRCT.

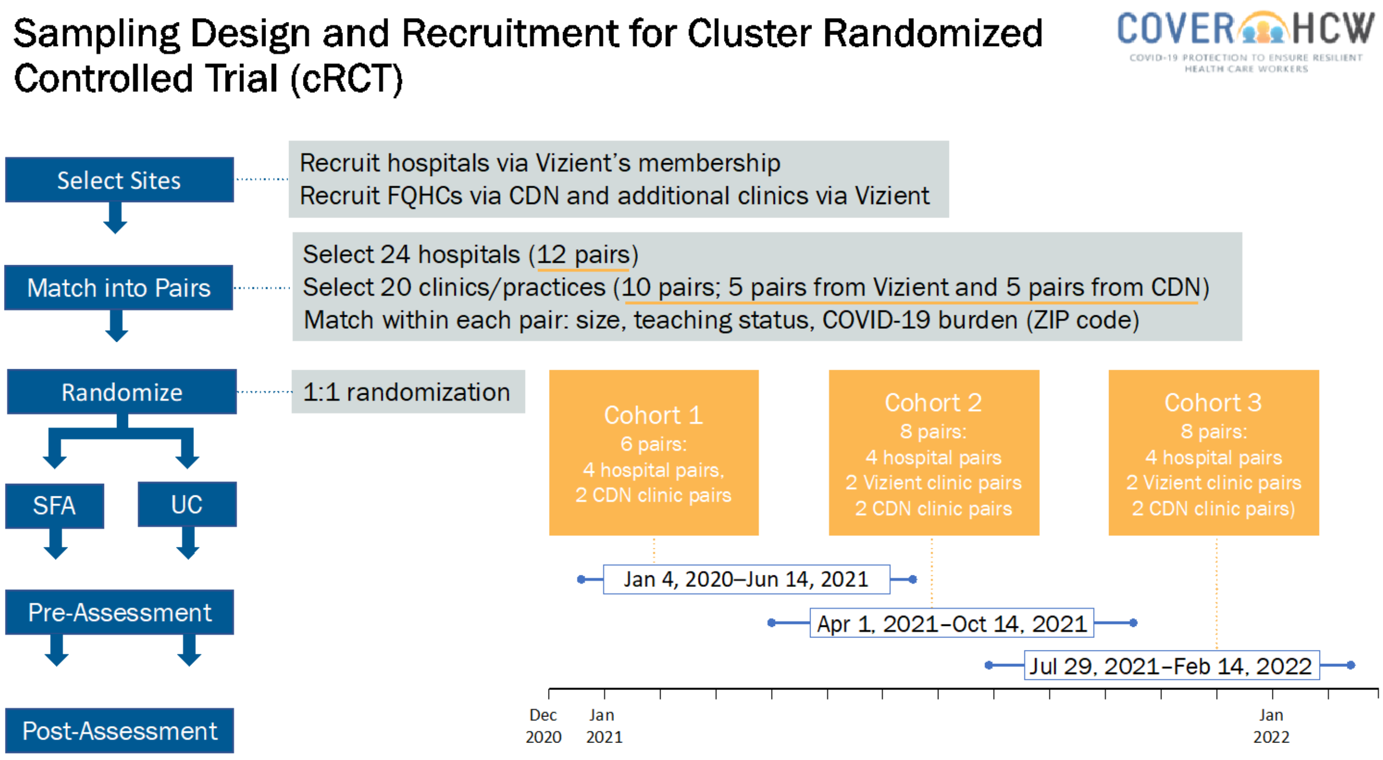

Exposures and Outcomes

Exposure. Our primary explanatory variable of interest is whether or not HCWs in the sites randomized to the intervention condition received the SFA training.

Primary Outcomes. Our primary outcomes include and acute PTSD symptoms of distress (e.g., Posttraumatic Stress Checklist, or PCL-5).[1] and chronic symptoms of general psychological distress (e.g., Kessler 6, or K-6) [2].

Secondary Outcomes. We are also including depression, anxiety, fatigue, sleep disturbance, and social roles/activities from scales such as PROMIS;[3, 4] burnout (using a single item with 5 response options ranging from 1 "I enjoy my work. I have no symptoms of burnout." to 5 "I feel completely burned out and wonder if I can go on. I am at a point where I may need some changes or may need to seek some sort of help.");[5] moral distress[6] (e.g., Moral Distress Thermometer); and resilience (e.g., Connor-Davidson Resilience Scale).[5, 7, 8] We are also collecting information about COVID-19 experiences and demographics (including HCW subgroup or role).

The full list of outcome measures in the survey, as well as secondary patient outcomes from Vizient and recruited hospitals/clinics as feasible are shown in Table 1 below.

Table 1. Outcome Measures

Measure	Description	# Items
**Primary Outcomes (survey)**		
Posttraumatic Stress Checklist (PCL-5)	DSM-5 measure of PTSD symptoms rated on 0-4 frequency scale	20
**Kessler 6 (K-6)**	General psychological distress	6
**Secondary Outcomes (survey)**		
PROMIS Sleep Related Impairment	PROMIS-29 subscale; items rated on a 1-5 scale from not at all to very much	4
Workplace stress	American Psychological Association's Stress in the Workplace survey; items rated on a 1-5 scale from strongly disagree to strongly agree	3
Burnout	Dolan single item burnout inventory; participants choose the description of burnout level that reflects their experience	1

Connor-Davidson Resilience Scale (CD-RISC-2)	Brief version the CD-RISC; items rated on a 1-5 scale from not true at all to true nearly all the time	2
Moral Distress Thermometer	Sliding scale with responses rated on a 0-10 scale from none to worst possible	1
**Health Care Worker Characteristics (survey)**		
COVID-19 Experience	Adapted from current COVID-19 surveys (American Life Panel, NIH, Shanafelt)	9
Demographics	Age, gender, race, ethnicity	4
Professional Characteristics	Provider type, years working at hospital/clinic	2
**Secondary Patient Outcomes (secondary data)**		
Patient quality and safety measures	Mortality, avoidable safety events (e.g., sepsis, pressure ulcers)	TBD
Patient experience of care	Patient experience: HCAHPS [9] for hospitals and CG-CAHPS for clinics, length of stay	TBD

We also include a list of interview topics/questions (Table 2).

**Table 2. Topics and Sample Questions for Interviews with Leaders, Champions, and HCWs**	
**Topic**	**Sample Question**
**Pre-Intervention Leaders**	
Sources of Support	What kinds of initiatives did you have in place to support health care workers in your facility before COVID-19?
Communication with Staff	Do you have a way for health care workers to provide feedback to voice concerns, either in general or with respect to safety and wellbeing?
COVID-19-related Challenges	What have been significant disruptions to work processes during the COVID-19 pandemic in your facility?
**Post-Intervention Leaders**	
**Facility-Level Factors (SFA Sites)**	What kinds of infrastructure changes were needed to accommodate the intervention?
**Sources of Support (UC Sites)**	In the past year, has your facility adopted any additional services or support systems to help health care workers address stress?
**General Impressions of Staff Health**	What are your general impressions of how health care workers are managing their stress/stressful situations now?
**Champions**	
Engagement with SFA	Which strategies did you use to encourage your fellow HCWs to join the intervention?
SFA Training	What did you find was particularly useful about the SFA training?<br>How could the training be improved?
CFIR Implementation factors e.g., adaptation, patient needs, hospital/clinic culture, leadership engagement	How did you adapt SFA for your setting?<br>How much demand was there for the training?<br>What support did you receive from [site name] to help facilitate SFA?
**HCWs**	
COVID-19-related Challenges	How would you describe the impact of COVID-19 on your role as a ___ at [site name]?
Motivation (SFA Sites)	What motivated you to participate in the SFA intervention?
Importance of HCW health and well-being	In general, how much do you feel [site name] emphasizes your health and wellbeing?
Impact of Core SFA Actions (SFA Sites)	Have you used a core SFA actions after a stressful instance?
Peer Support	How do you and your fellow HCWs help each other out during stressful times?

Intervention and Comparator

We will compare the impact of SFA to UC on HCW mental and physical well-being. In the SFA condition, we will identify 2-4 champions depending on participating site's needs for cross-coverage and to increase sustainability. We will implement SFA using a "train-the trainer" model. We anticipate that existing or planned interventions in UC sites could dampen the detection of the effect of the SFA intervention.[10] We will address this by carefully documenting the types of activities being implemented before and after the SFA intervention and conduct post-hoc analyses to further examine variation across sites in comparing SFA with UC. Even in the case of a null result, we will have assembled an incredibly rich, national qualitative data set across multiple settings and HCW types for an in-depth understanding of strategies to address HCW mental well-being.

Masking (or blinding) of research staff will be performed to the extent possible in this study. The survey data can be analyzed in a blinded fashion. However, due to the nature of qualitative research (described below), masking will not be possible for this data collection effort. Given that no qualitative research can be performed in a blinded way, we do not anticipate the lack of masking to affect our qualitative results adversely.

Implementation of Intervention

Champions will receive the two-hour training and materials, and develop a plan to train HCW teams locally. We considered recruiting only certain clinician types, but discussions with partners and national organizations (American College of Emergency Physicians) underscored the importance of training all members of HCW teams (e.g., physicians, nurse practitioners, nurses, respiratory therapists, medical assistants) to avoid disrupting important relationships and leverage existing peer support.

Once trained, the champions will take their training plan back to their organizations to begin training their peers. Each hospital is expected to recruit an average of 170 HCWs, which can be spread across multiple teams or units (e.g., ICUs, ED, general floors, specialty services, etc.). Ambulatory practices are expected to recruit an average of 50 individuals, which may include both HCWs and supporting staff who are patient-facing (e.g., front desk staff). The selection of teams and units is expected to be made by the site leaders and champions together. The champions will have some flexibility in how the training is implemented, to ensure that local context and other important factors are taken into account.

While the intervention sites implement SFA, the control sites will be expected to continue their usual care (meaning whatever programs had been in place or not for HCWs). The control sites will be asked only to support the survey of HCWs in analogous teams and units compared to their paired intervention site. For example, if the intervention site is imeplementing SFA in their ICUs, the control site will be asked to field the survey to similar HCWs in their ICUs.

Data Collection and Safeguarding

Our clinical partners will collect names and email addresses for potential study participants (HCWs, site leaders, and site champions) during recruitment. No patients will participate in this study. This identifying information will be transmitted to RAND from external organizations via secure file transfer protocol, e.g., Kiteworks. These data will be stored on a secure RAND server, and accessible only by authorized project personnel.

We will only use identifying information to administer the web- based surveys, schedule interviews, and administer continuing education credits for individual participation in training. No response data will include any identifying information other than the facility type and professional type (in a sufficiently granular was so that individuals cannot be identified by attribution). Individuals will be assigned an anonymous study identifier to link the pre- and post-surveys for analytic purposes. Interview responses will also remain confidential by only noting the facility type and respondent type.

Survey Data. A brief online survey of HCWs will be conducted pre- and post-intervention in both SFA and UC conditions and will include measures of physical and psychological symptoms determined to be of importance to this population. Primary outcomes collected by the survey include PTSD (e.g., Posttraumatic Stress Checklist, or PCL-5) and general psychological distress (K-6). Secondary outcomes collected by the survey include depression, anxiety, fatigue, sleep disturbance, and social roles/activities from scales such as PROMIS; burnout (e.g., with the Dolan single item); moral distress (e.g., Moral Distress Thermometer); and resilience (e.g., Connor-Davidson Resilience Scale). We will also use the surveys to collect HCW characteristics such as COVID-19 experiences and demographics.

The survey will be administered by the RAND Survey Research Group (SRG). SRG will send individual e-mail invitations to respondents at participating hospitals and clinics, asking them to participate in the online survey. The list of physician emails to use in these invitations will be provided by contacts the participating hospitals and clinics. Each individual will receive a custom URL link allowing us to identify respondents from non-respondents for follow-up purposes.

The survey will be voluntary. Informed consent will be implied if they complete the survey, which should take about 20 minutes. SRG will have individual identifiers so that we can follow-up with non-respondents. However, SRG will strip these identifiers from the survey data prior to giving to the RAND research team for analysis.

All data collection and Record Management System (RMS) functions will be conducted on SRG's secure network segment. Computers on the secure network segment are isolated from the rest of the RAND network (e.g. no Internet access, e-mail or file sharing between these computers and the unclassified network) minimizing the possibility of infection by malicious software and unintentional exposure of sensitive data. The computers on the segment will also employ standard password protection along with file and folder permissions limiting access to appropriate project staff. All servers on the secure network segment are in the RAND Data Center and all client computers are located in offices with limited OmniLock access. Within the segment the RMS database containing the sample data uses a separate password to restrict access. The RMS server is not backed up by IST but backed up daily by SRG. The backup files are encrypted and then placed in on a separate server that is subject to IST backups.

Survey data will be collected in ConfirmIt, a survey software tool. The Confirmit platform is hosted on dedicated servers in a Rackspace data center in the United States. With ConfirmIt, data is protected at rest and in transit by strong cryptographic encryption; administrative access to systems by Confirmit staff is regulated by multi-factor authentication. Additionally, customer security controls include password complexity, lock out, one-way hashes, and multi-factor authentication (MFA). For this study, the SRG programmers will have the ability to author surveys and load/retrieve data from ConfirmIt. RAND has enforced the use of PGP encryption that provides RSA level security. All RAND users of Confirmit use Confirmit's MFA using Google Authenticator to administer, author, and extract data from Confirmit.

HCW response rates may be a challenge, but eliciting outcomes directly from HCWs is critical. HCWs will receive $25 for completing the survey, which will be endorsed by site leaders and champions. Lack of data due to low response is also mitigated by extensive qualitative data. Of note, we will only use identifying information to administer the web-based surveys (and for scheduling the interviews, see qualitative data collectiob below). No response data will include any identifying information other than the facility type and professional type. Individuals will be assigned an anonymous study identifier to link the pre- and post-surveys for analytic purposes. Interview responses will also remain confidential by only noting the facility type and respondent type (Leader, Champion, specialty).

Interview Data. We will collect in-depth qualitative data using standardized interview protocols to understand how the well-being of HCWs is impacted by the workplace environment. This qualitative exploration will shed light on additional, unforeseen factors that health care system leaders and HCWs see as important to shaping their well-being. In addition, interviews with health care system leaders will aid in understanding how particular contextual workplace environmental factors could intersect with the implementation and impact of the intervention, in turn ensuring that we can provide a more complete picture of the intervention's effectiveness and impact on HCWs. We will also interview site Champions following the SFA training at the local hospital/clinic/FQHC to learn about implementation experiences.

Informed consent will be gathered by all interview participants in accordance with Institutional Review Board (IRB) determinations. To ensure confidentiality, the contact information of interview participants will be password-protected and stored on encrypted RAND computers. This file will be destroyed upon finalization of data analysis. Any names and other identifying details will be removed from interview transcriptions, so as to not be incorporated into the data analysis and subsequent reporting.

All interviews will be audio recorded and detailed notes will be taken. Notes will be scrubbed of any identifiable information. Recordings and notes will be stored on encrypted RAND computers and transmitted for transcription through the secure file sharing platform, Kiteworks. Once the interviewer proofreads the notes for accuracy, completion and confidentiality, consulting the recording as needed, the notes will be uploaded into Dedoose, anencrypted qualitative data analysis software program, which facilitates inductive and deductive analysis. Upon completion of the research, all audio files and written notes will be destroyed.

Secondary Data. We will obtain low-burden, routinely collected, site-level, patient-related outcomes pre- and post-

intervention as feasible. Vizient's hospitals submit safety and quality measures, including mortality, length of stay, and safety events, while CDN does not. While Vizient and CDN do not routinely collect patient experience data, we will ask sites to share aggregate pre- and post- scores on patient experience surveys such as HCAHPS[9] (hospitals) and CG-CAHPS[11] (clinics). Sites may suspend collection of CAHPS data during the pandemic, but our partners believe that many will still collect some patient experience data.

Responsibility for Data Safeguarding. Drs. Meredith and Gidengil will share overall responsibility for the safeguarding of all data under RAND's control and will have primary responsibility for familiarizing all the project staff with this data safeguarding plan and procedures. They, with support from Ms. Dalton, will follow up with project staff at periodic intervals to ensure that they are in compliance with these data safeguarding procedures.

Data Sensitivity. Data to be collected will include aggregate measures of patient safety and satisfaction and individual characteristics including personal identifiers (name, email addresses), demographics (age, gender, race/ethnicity), professional characteristics, and measures of physical and psychological symptoms.

Data Transmittal and Storage. Project partners will provide names and contact information for staff at recruited hospitals, FQHCs, and clinics. Project partners and participating hospitals, FQHCs and clinics will also provide aggregate secondary data files. This information will be transmitted to RAND from these external organizations via secure file transfer protocol, e.g., Kiteworks. These data will be stored on a secure RAND server and accessible only by authorized project personnel.

For survey administration, contract information will be transmitted to SRG via secure file transfer protocol, e.g., Kiteworks, and stored on SRG's secure network segment.
Recordings and notes from all interviews will be stored on encrypted RAND computers and transmitted for transcription through the secure file sharing platform.

Disclosure Risks. Participation in the study may pose direct risk for disclosure of sensitive information. While we will only report data aggregated to the site-level using anonymized facility codes, the release of individual responses through an unlikely breach of confidentiality about HCWs could potentially lead to embarrassment or negative consequences for their reputation and career.

Audit and Monitoring Plans. Drs. Meredith and Gidengil, with support from Ms. Dalton, will follow up with project staff at periodic intervals to ensure that they are in compliance with these data safeguarding procedures.

Destruction of Data. Identifiable data will be destroyed upon project completion, no later than November 31, 2023. Fully de-identified data will be stored indefinitely by creating a new data set. This new data set will only include non-identifiable variables collected through the surveys, interviews, and contained in secondary data files as well as derived variables (those created by RAND based on variables obtained through this project). None of the retained variables will be linked to names or any variables containing contact information.

Please see the *Data Saftguarding Plan* for additional details.

Analytic Methods

Quantitative Evaluation (Aim 1)

We will test the hypothesis that HCWs in the sites that receive SFA training will have better mental and physical well-being compared with HCWs in UC sites. We will apply a difference-in-differences (DID) model, which is robust to unobserved differences at the baseline and temporal trends unrelated to treatment. Let t=0,1 represent the baseline and follow-up waves, and D represent the treatment status (D=1 for SFA and D=0 for UC). Let Y_{ijt} be the observed outcome for HCW i in clinic j at time t. The DID model is $Y_{ijt} = \alpha + \beta \times I\{t=1\} + \gamma D + \lambda \times D \times I\{t=1\} + X_{ij}\theta + \varepsilon_{ijt}$. In this model, α is the intercept, β is the temporal trend not related to the intervention, γ is the mean difference in the outcome at

baseline adjusted for individual-level covariate effects $X_{ij}\theta$. The random error ε_{ijt} is correlated within a respondent and possibly within the same clinic. The hypothesized treatment effect is represented by the interaction effect λ, which is tested by the regular Wald's z-test using robust sandwich standard error to adjust for intra-class correlation. We will fit the model by the generalized estimating equation (GEE) method. Depending on goodness of fit to real data, we may choose a multiplicative scale, e.g., odds ratio, to implement the DID analysis.

Subgroup Analyses. We will also be able to conduct two subgroup analyses (for the hospital and clinic subgroups). The two subgroup analyses are completely non-overlapping and share the same approach and outcome measures. Despite the potentially different effect sizes, the statistical analysis for the overall effect assessment and the two subgroup analyses will follow the same DID modeling method. We will also fit a three-way interaction among facility type, treatment status, and time, to combine the two subgroup models into a single model, i.e., a difference-in-differences-in-differences (DDD) model to partially improve statistical power, if the two subgroup DID analyses exhibit a similar level of measurement errors. If a generalized linear model (GLM), e.g., a logistic regression, is adopted in subgroup analyses, we will not fit a DDD model since there will be no notable gain in asymptotic efficiency by a DDD model in this setting.

Missing Data. We anticipate that missing data could happen at multiple levels. First, as stated in the power analysis and analytic plan, many sites and respondents will not respond to our survey request at all. These unit-level nonresponses can result in a systematic difference between the treated and non-treated subsamples. However, the DID approach can eliminate such a time-invariant bias from the estimation, if present. The reduction in sample size and statistical power have also been calculated in the original proposal. Second, our online survey tools will ensure that respondents must fill in all items in measurement instruments. Thus, we will not have the issue of item-level missingness among respondents. Third, we acknowledge that some respondents in the pre-intervention wave may not complete the post-intervention survey request, who are referred to as partial respondents. We will examine the baseline differences between partial respondents and the complete respondents who have both waves of data. We will conduct two types of analysis: a complete-case analysis and an imputation analysis. In the complete-case analysis, we will run all statistical models for complete respondents only. In the imputation analysis, we will fit an imputation model by complete respondents, using the baseline characteristics and the observed pre-intervention data to predict the post-intervention data. We will impute the missing values of the partial respondents by the fitted imputation model. We plan to run 20 rounds of imputations and aggregate the results by standard multiple imputation process. We will report results from both the imputation and complete-base analyses and acknowledge the specific limitations in each type of analysis.

Sample Size, Power, and Analytic Plan. Assuming a 40% response rate for hospitals (24-30 sites with 170 HCWs each) and 50% for clinics (20-24 sites with 50 HCWs each), depending on number of recruited hospitals and clinics we expect between 2,132 and 2,520 completed surveys at each of pre- and post- intervention. We calculated the effect sizes that can be detected under the regular setting of two-sided p-value <.05 and power >.8 in a two-sample comparison for the PCL-5 measure of PTSD symptoms. For the PCL-5, a minimally important difference (MID) is 5-10 points and a clinically significant change is 10-20 points.[1] We considered a range of sample sizes corresponding to different site-level and individual-level response rates. We also calculated the overall effects pooling all samples and the subgroup effect within a health care setting using a part of the sample. We have hypothesized potentially differential treatment effects between hospitals and clinics/FQHCs. Hospitals are larger with more staff distributed across multiple departments, whereas the clinics/FQHCs are much smaller and all staff are in a single location. HCWs in clinics will also be at lower risk given more care is being practiced telephonically. Therefore, conceptually types of facility may moderate the treatment effect.

We assumed an intra-class correlation (ICC) of 0.01 within each site. Table 1 lists the minimal detectable effect sizes under the assumptions above. In summary, the planned sample size is more than sufficient to detect a small effect for both the overall evaluation and subgroup analysis (see below) for the hospital sample even under substantial nonresponses at both the site and respondent level. The detectable effect size is moderately small for the clinic

Table 1. Detectable effect sizes in the unit of standard deviation of an outcome under different sample size

Hospital (# sites × # respondents)	Clinic (# sites × # respondents)	Total sample size	Overall effect size	Subgroup effect size
30 × 68	24 × 25	2520	0.14	0.16 (hospitals), 0.26 (clinics)
24 × 68	20 × 25	2132	0.15	0.18 (hospitals), 0.28 (clinics)
24 × 48	20 × 20	1552	0.17	0.20 (hospitals), 0.31 (clinics)

sample due to its smaller sample size. For example, under the planned sample size (24 hospitals × 68 respondents, 20 clinics × 25 respondents), we can detect an overall effect of 0.15 SD, an effect of 0.18 SD for hospitals, and 0.28 SD for clinics. These standardized effect sizes translate to 0.83, 0.99, and 1.54 points for the PCL-5 score (SD≈5.5), respectively, all of which are either below or around the MID. If the cluster-level response rate allows, we will consider randomization stratified by geographic areas.

Qualitative Evaluation (Aims 2 & 3)

Our interview protocols rooted in the Consolidated Framework for Implementation Research (CFIR)[12] and developed in partnership with our working group. An in-depth qualitative exploration is important for understanding how the well-being of HCWs is impacted by the workplace environment. This qualitiative exploration will shed light on additional, unforeseen factors that health care system leaders, intervention champions, and HCWs see as important to shaping their well-being. In addition, interviews with health care system leaders will aid in understanding how particular contextual workplace environmental factors could intersect with the implementation and impact of the intervention, in turn ensuring that we can provide a more complete picture of the intervention's effectiveness and impact on HCWs.

We will base the evaluation questions on the CFIR, a a rich tool with which to assess workplace environment factors, such as organizational culture, networks and communications, perceived self-efficacy, available resources, et cetera. Drawing from the existing literature, we will identify both workplace contextual factors (i.e., organizational-level factors such as climate, culture, practices, resources) [13, 14] and other contextual factors influenced by the workplace (i.e., job-level factors such as roles and requirements) that play an important role in predicting worker well-being, including mental health and burnout outcomes. Such factors are likely to be particularly important in health care environments in which HCWs operate under high-stakes conditions, work closely with each other in interprofessional teams, adhere to entrenched job hierarchies and role boundaries, and contribute significant cognitive, physical, and emotional investment to ensuring high-quality patient outcomes.[15, 16] Thus, existing workplace contextual factors that affect to HCW well-being under normal circumstances are likely to be further accentuated during a pandemic.

Prior work has indicated that performance and well-being of HCWs is affected by macro-level factors (health care system) as well as micro-level factors (units within the system, which may be clinics, emergency departments, intensive care units) that will vary across the health care systems and across units within the health care systems.[17, 18] We will explore several characteristics at both the system- and unit-level through our hospital leadership interviews, champion interviews, and HCW survey.

At the system level, we will assess: 1) supportive leadership (as reported by leaders and perceived by champions), and 2) organizational culture (safety; professional growth; equity; mental health care availability and support) as key workplace factors. At the unit level, we will examine: 1) role clarity, 2) team cohesion as relevant workplace factors that may affect HCW well-being, and 3) for those HCW receiving SFA, how they perceived the overall effectiveness of the intervention. Thus, this portion of the work will incorporate an inductive and deductive ethnographic approach that accounts for existing theories on intervention implemention (e.g., CFIR) while allowing for the discovery of novel, context-specific factors that are emerging during this unprecendented public health crisis.

We will ensure that workplace context factors are considered extensively through all modes of data collection as below:

(1) ***HCW survey (pre- and post-intervention)***. We will include validated item sets for key workplace environment factors in our HCW survey pre-intervention, including composite items (e.g., Teamwork, Staffing and Workplace) from AHRQ's Surveys on Patient Safety Culture™ (SOPS®).[19, 20] These surveys have been developed for multiple health care settings, including hospitals and medical offices.

(2) ***Health Care system leader interviews (pre- and post-intervention).*** We will conduct brief, structured interviews will health care system leaders. During the interview, we will incorporate questions from the AHRQ Survey on Patient Safety Culture. In addition, will discuss policies around advancement and professional development, and existing services for HCW mental health to understand the support offered by leadership to HCWs. Having

leaders complete the relevant portions of the AHRQ Survey on Patient Safety Culture in advance of the interview will help refine follow-up questions within the interview, thereby reducing the burden on leaders.

(3) ***Intervention champion and HCW interviews (post-intervention)***. We will also include questions about these workplace factors in our interviews with champions and HCWs. As part of our semi-structured interview guide, we will explore system-level perceptions of supportive leadership and organizational culture (including safety, opportunities for professional growth, and equity/fairness). At the unit level, we will specifically ask about role clarity and team cohesion.

Given the above plan to explore workplace contextual factors in depth, and specifically their impact on HCW well-being and interaction with and effect on the SFA intervention, we believe this work is ideally poised to deepen the collective understanding of such factors.

The interview procedures are as follows: We will conduct interviews up to a specified N within each group until we have reached thematic saturation.[21] ***Pre-intervention***, we will perform brief structured interviews with at least one *leader* (e.g., Chief Quality Officer) at SFA and UC sites (N~44) on: (1) organizational-level characteristics (e.g., % of patients with COVID-19, HCW absenteeism); (2) interventions in place to support HCW well-being; and (3) anticipated facilitators and barriers to implementation, particularly in light of COVID-19 (SFA sites only). Leaders will be asked to speak on behalf of their site. In order to account for all salient organizational-level characteristics, exposure to exisiting strategies to address HCW well-being, and anticipated facilitators and barriers, we will sample from each site. As part of the structured nature of the interview, we will adapt and administer questions from the AHRQ Survey on Pateint Safety Culture, probing leaders to elaborate on certain responses as needed.

Post-intervention, we will conduct brief, semi-structured interviews with *champions* (N~46) at all SFA sites for insights into factors to consider when refining and then scaling up the intervention. Sampling champions across all SFA sites is important for eliciting the full range of the on-the-ground perspectives on SFA implementation and actions taken to drive uptake and sustainment of SFA. Champions will also be asked to elaborate on the unit- and health care system-level factors they feel would need to adjust to optimize the implementation of SFA.

We will also perform semi-structured interviews among a range of *HCWs* (N~132), or until thematic saturation is reached, at all sites to understand the SFA/UC impacts, COVID-19-related stress and trauma, sources of support, role clarity, and perspectives on the effectiveness of strategies to improve HCW well-being. Working with our health care systems partners, we will purposively sample ~2-3 representatives across HCW types at all sites for a richer understanding of differential effects across various types of HCWs (e.g., respiratory therapists, nurses, physicians).

Finally, we will conduct brief exit interviews with *health care system leaders* (N~44) at UC and SFA sites. These exit interviews with site leaders will round out an analysis of organizational-level factors that drove the effectiveness of the intervention and the well-being of HCWs more broadly.

Informed consent will be gathered by all interview participants in accordance with Institutional Review Board (IRB) determinations. To ensure confidentiality, the contact information of interview participants will be password-protected and stored on encrypted RAND computers. This file will be destroyed upon finalization of data analysis. Any names and other identifying details will be removed from interview transcriptions, so as to not be incorporated into the data analysis and subsequent reporting.

All interviews will be audio recorded and detailed notes will be recorded. Notes will be scrubbed of any identifiable information. Recordings and notes will be stored on encrypted RAND computers and transmitted for transcription through the secure file sharing platform, Kiteworks. Once the interviewer proofreads the notes for accuracy, completion and confidentiality, consulting the recording as needed, the notes will be uploaded into Dedoose[22], a encrypted qualitative data analysis software program, which facilitates inductive and deductive analysis.[23-25] Upon completion of the research, all audio files and written notes will be destroyed.

The procedures for qualitative data analysis are as follows: the qualitative evaluation lead will consult with the team leads to draft preliminary codebooks for each type of interview (site leadersn pre- and post-intervention, champions, and HCWs). The codebooks will map to focus group and interview protocols that received approval from the IRB. In addition, the codebooks will incorporate key aspects of the CFIR.

The qualitative evaluation lead (will work closely with a research assistant with extensive qualitative data analysis experience to collectively read through a sample (~10) of each set of interviews to identify additional relevant codes. The team lead will independently review a select number of transcripts within Dedoose to test the structure of the coding scheme (e.g., identification of themes and exemplars). To ensure rigor, transparency, credibility, reliability, inter-rater reliability (IRR) of the coding across the qualitative data analysis team will be evaluated with a Cohen's Kappa statistic. When inter-rater reliability falls below .8, raters will review discrepancies and come to consensus. Cohen suggested the Kappa result be interpreted as follows: values ≤ 0 as indicating no agreement and 0 .01–0 .20 as none to slight, 0 .21–0 .40 as fair, 0 .41– 0 .60 as moderate, 0 .61–0 .80 as substantial, and 0 .81– 1 .00 as almost perfect agreement.[26]

As the coding team examines the transcripts, they will follow Butler-Kisber's[27] approach by follow two core stages of analysis, beginning with a coarse-grained phase. Actions in this stage include reading and rereading, dialoguing about what is being revealed, and/or writing reflective memos to broadly classify emerging themes. The second-phase of analysis, the fine-grained phase, is when the researchers examine the pieces of data more closely. These "chunks" of data are "reassembled into more refined categories, and are broken down into others, and these are assigned, and reassigned names or codes."[27] The second phase of analysis isolates specific words, phrases, and ideas that represent larger themes. These more specific, discreet units of data generate support for identified abstract concepts or ideas.

After completing the coding process, we will develop qualitative and quantitative results to describe the data. Expected outputs include: Code frequency counts; Code co-occurrence (i.e., overlap between codes); Descriptor (e.g., size of a site; burden of COVID-19) x codes; Deviant case analysis to document instances of atypical workplace environmental factors and perspectives; and Summarized, qualitative overviews of key themes (including exemplar direct quotations) that map to each set of interviews. We will stratify themes by sub-sample to examine the distribution of themes across types of HCWs, site type and size (e.g., hospital versus clinic), region, et cetera, as well as the identification of cross-cutting themes to refine the intervention tools and guide implementation. To enhance credibility, we will cross-check these findings with key representatives among our partners. Ultimately, the qualitative data will provide rich insights on how to account for workplace environmental factors when evaluating the patient-centered effectiveness of SFA and issues to consider for scale-up.

Human Subjects Protection

IRB. To esure protection of human subjects, the project's study protocol and evaluation materials have been review and approved by the RAND Human Subjects Protection Committee (HSPC). The HSPC serves as RAND's Institutional Review Board (IRB) and is charged with ensuring the ethical treatment of all individuals who are participants in any RAND projects through observation, intervention, interaction or use of data about them. RAND's "Federalwide Assurance for the Protection of Human Subjects" (FWA00003425, effective until June 22, 2023) serves as assurance of compliance with the regulations of 16 federal departments and agencies. Any meaningful changes to the protocol or materials must be submitted to HSPC for review and approval prior finalization.

When multiple institutions involved in a single study are engaged in human subjects research requiring IRB review, duplicate reviews can be avoided by having one institution defer review to the other. For this study, participating hospitals, clinics, and FQHCs may conduct independent review or defer review to RAND's HSPC by executing a study-specific IRB Authorization Agreement (IAA).

Adverse Events. Our study intervention is inherently low-risk. There are no physical risks to participants in this study, and psychological risks are primarily related to the sensitivity of some of the measures (e.g., PTSD and depression

symptoms). Therefore, adverse events (AEs) experienced by study subjects are likely to be due to the participants' underlying mental illness, and/or high-risk situations associated with COVID-19 surge, rather than the intervention itself. Any emergency identified by hospital or clinic/center staff will be handled using existing emergency procedures that are already in place for clinical staff.

An AE is when a study participant reports to a member of the research team that they are having thoughts of suicide or self-harm, or thoughts of harming someone else, but where there is no imminent danger. For example, a statement like, "I have thought about killing myself." or "If I go home, I could overdose." would be considered an adverse event. The research team member may need to ask follow-up questions to determine how risky the situation is, and whether there is imminent danger—defined as the person having a specific plan, means to carry out the plan, and the intention of carrying out the plan. AEs may be identified while conducting interviews with HCWs, Leaders, and Champions. Interviewers will be asked to report these situations to the study MPIs.

A serious adverse event (SAE**)** is when a study participant discloses to a member of the research team that they are having thoughts of suicide or self-harm, and where the participant is thought to be in imminent danger. It also includes any situation where a participant reports child or elder abuse, or the intention to harm someone else, or when the research team learns that a patient has died or has been hospitalized for a study-related condition. For example, a participant who says they have a gun and are thinking about killing themselves or someone else, The research team member may need to probe further by asking follow-up questions using the Columbia-Suicide Severity Rating Scale (CSSRS) to determine the acuity of the risk (see below). If a participant says their child is being physically abused by a family member, the research team member must report it by calling the National Child Abuse Hotline (see below). AEs may be identified while conducting interviews with HCWs, Leaders, and Champions. Interviewers will be asked to report these situations to Dr. Meredith and Gidgengil.

DSMB. We have established a Data Safety and Moniring Board (DSMB) to monitor HCW safety throughout the entire course of the intervention and assessment period, specifically during the period that involves pre-intervention assessment, intervention delivery, and follow-up assessment for each of the three cohorts over a 14-month period. The DSMB includes experts in scientific disciplines needed to monitor the data and ensure HCW safety during the conduct of this study, including an emergency medicine physician, a psychiatric epidemiologist, and a psychiatrist/trialist. DSMB members have no formal role on the project and no conflicts of interest with study outcomes. DSMB procedures conform with *PCORI's Policy on Data Safety Monitoring Plans for PCORI-Funded Research* consistent with the following principles to:

- Operate independently and without undue influence from any interested party, including study investigators, other members of the research team such as patient research partners, and PCORI staff;
- Protect the interests of research study participants and ensure that they are not exposed to undue risk;
- Have access to data throughout the course of the study and interim analyses of research study data.
- Allow access to unmasked data only to DSMB members and a small group of additional team members (the MPIs, SRG staff, and the study statistician) overseeing the analysis; and
- Access to external evidence, such as recent related publications, that could inform the research study.

The main responsibilities of the DSMB include, but are not limited to the following: (1) reviewing the research protocol, consent form(s), and plans for data and safety monitoring prior to the initiation of the study; (2) monitoring of the progress of the study, including data quality, timeliness, recruitment and retention of study participants, AEs, SAEs, reasons for participant withdrawal, adherence to the timeline of the study, protocol deviations and violations, performance across study sites, and factors that may affect the risks and benefits of the study such as emerging literature; and (3) making directives about the continuation, modification, or termination of the study, based on the balance of adverse events and beneficial outcomes with the assistance of the study statistician. Throughout the study, notification of SAEs as well as any proposed investigator-initiated changes in the protocol will be submitted to the DSMB. Based on its review of the protocol, the DSMB will identify the data parameters and format of the information to be reported on a regular basis. The DSMB may at any time request additional information from the MPIs. All SAEs and AEs will only be reported to the DSMB at each of the four planned meetings. Events will be tabulated and submitted to the DSMB. Based on review of safety data, the DSMB will issue directives concerning the

conduct of the study. Recommendation/directives made by the DSMB may include amending safety monitoring procedures, modifying the protocol or consent, terminating the study, or continuing the study as designed.

References

1. The PTSD Checklist for DSM-5 (PCL-5) (US Department of Veterans Affairs) (2013).
2. Prochaska JJ, Sung H, Max W, Shi Y, Ong M. Validity study of the K6 scale as a measure of moderate mental distress based on mental health treatment need and utilization. Int J Methods Psychiatr Res. 2012;21(2):88–97. doi: 10.1002/mpr.1349
3. HealthMeasures. Why use PROMIS®. Northwestern University. Accessed March 25, 2019. http://www.healthmeasures.net/explore-measurement-systems/promis
4. U.S. Department of Health and Human Services. PROMIS®-29 Profile v2.0 Adult Profile©. National Institutes of Health (NIH). Accessed March 18, 2019. www.assessmentcenter.net
5. Dolan ED, Mohr D, Lempa M, Joos S, Fihn SD, Nelson KM, Helfrich CD. Using a single item to measure burnout in primary care staff: a psychometric evaluation. J Gen Intern Med. May 2015;30(5):582-7. doi:10.1007/s11606-014-3112-6 PMC4395610
6. Wocial LD, Weaver MT. Development and psychometric testing of a new tool for detecting moral distress: the Moral Distress Thermometer. Journal of Advanced Nursing. 2013;69(1):167-174.
7. West CP, Shanafelt TD, Kolars JC. Quality of Life, Burnout, Educational Debt, and Medical Knowledge Among Internal Medicine Residents. JAMA. 2011;306(9):952-960. doi:10.1001/jama.2011.1247 %J JAMA
8. Vaishnavi S, Connor K, Davidson JR. An abbreviated version of the Connor-Davidson Resilience Scale (CD-RISC), the CD-RISC2: psychometric properties and applications in psychopharmacological trials. Psychiatry Res. Aug 30 2007;152(2-3):293-7. doi:10.1016/j.psychres.2007.01.006
9. Agency for Healthcare Research and Quality. CAHPS ECHO survey measures. AHRQ. Accessed March 18, 2019. https://www.ahrq.gov/cahps/surveys-guidance/echo/index.html
10. Macklin R, Natanson C. Misrepresenting “Usual Care” in Research: An Ethical and Scientific Error. The American Journal of Bioethics. 2020/01/02 2020;20(1):31-39. doi:10.1080/15265161.2019.1687777
11. Agency for Healthcare Research and Quality. CAHPS Clinician & Group Survey. AHRQ. Accessed May 20, 2020. https://www.ahrq.gov/cahps/surveys-guidance/cg/index.html
12. Damschroder LJ, Aron DC, Keith RE, Kirsh SR, Alexander JA, Lowery JC. Fostering implementation of health services research findings into practice: a consolidated framework for advancing implementation science. Implementation Science. 2009/08/07 2009;4(1):50. doi:10.1186/1748-5908-4-50
13. Shannon HS, Robson LS, Sale JE. Creating safer and healthier workplaces: role of organizational factors and job characteristics. Am J Ind Med. Sep 2001;40(3):319-34. doi:10.1002/ajim.1106
14. Shannon HS, Robson LS, Sale JE. Creating safer and healthier workplaces: role of organizational factors and job characteristics. American journal of industrial medicine. 2001;40(3):319-334.
15. McLellan RK. Work, Health, And Worker Well-Being: Roles And Opportunities For Employers. Health Aff (Millwood). Feb 1 2017;36(2):206-213. doi:10.1377/hlthaff.2016.1150

16. McLellan RK. Work, health, and worker well-being: roles and opportunities for employers. Health affairs. 2017;36(2):206-213.
17. Dieleman M, Harnmeijer JW. Improving Health Worker Performance: in Search of Promising Practices. 2006:77.
18. Dieleman M, Harnmeijer JW. Improving health worker performance: in search of promising practices. Geneva: World Health Organization. 2006:5-34.
19. About SOPS. Agency for Healthcare Research and Quality.
20. Surveys on Patient Safety Culture™ (SOPS®). Agency for Healthcare Research and Quality. Updated Content last reviewed June 2020. Accessed Aptil, 2020. https://www.ahrq.gov/sops/contact-us/index.html
21. Lowe A, Norris AC, Farris AJ, Babbage DR. Quantifying Thematic Saturation in Qualitative Data Analysis. Field Methods. 2018;30(3):191-207. doi:10.1177/1525822x17749386
22. Dedoose Version 8.3.35, web application for managing, analyzing, and presenting qualitative and mixed method research data (2020). . Los Angeles, CA: SocioCultural Research Consultants, LLC; 2020.
23. Bernard H, Ryan G. Analyzing qualitative data: Systematic approaches. Sage Publications; 2016.
24. Guest G, MacQueen K, Namey E. Applied thematic analysis. Sage Publications; 2011.
25. Miles M, Huberman A, Saldana J. Qualitative data analysis. Sage Publications; 2013.
26. McHugh ML. Interrater reliability: the kappa statistic. Biochem Med (Zagreb). 2012;22(3):276-82.
27. Butler-Kisber L. Qualitative inquiry: Thematic, narrative and arts-informed perspectives. Sage Publications; 2010.
