# Supplementary material for: Testing an Intervention to Improve Health Care Worker Well-Being During the COVID-19 Pandemic: A Cluster Randomized Clinical Trial
Source: JAMA Netw Open. 2024 Apr 30;7(4):e244192. doi: 10.1001/jamanetworkopen.2024.4192 (PMC11061774; doi:10.1001/jamanetworkopen.2024.4192)
Supplement: Supplement 2. — eTable 1. Survey Fielding Dates by Cohort eTable 2. Unadjusted Outcomes Pre- and Post-Intervention by Treatment Status for HCWs in FQHCs eTable 3. Unadjusted Outcomes Pre- and Post-Intervention by Treatment Status for HCWs in Hospitals eTable 4. Intention-to-Treat Results by Age Group for the Full Sample and by Facility Type [file jamanetwopen-e244192-s002.pdf]

Supplemental Online Content

Meredith LS, Ahluwalia S, Chen PG, et al. Testing an Intervention to Improve Health Care Worker Well-Being During the COVID-19 Pandemic: a Cluster Randomized Clinical Trial. *JAMA Netw Open*. 2024;7(4):e244192. doi:10.1001/jamanetworkopen.2024.4192

eTable 1. Survey Fielding Dates by Cohort

eTable 2. Unadjusted Outcomes Pre- and Post-Intervention by Treatment Status for HCWs in FQHCs

eTable 3. Unadjusted Outcomes Pre- and Post-Intervention by Treatment Status for HCWs in Hospitals

eTable 4. Intention-to-Treat Results by Age Group for the Full Sample and by Facility Type

This supplemental material has been provided by the authors to give readers additional information about their work.

© 2024 Meredith LS et al. *JAMA Network Open*

eTable 1. Survey Fielding Dates by Cohort

Cohort	Pre-Intervention Survey Dates	Post-Intervention Survey Dates
1	3/11/21-4/9/21 (4 sites)	6/14/21-7/16/21 (4 sites)
2	7/7/21-8/9/21 (4 sites)<br>8/30/21-10/4/21 (4 sites)<br>9/8/21-10/8/21 (2 sites)<br>10/6/21-11/16/21 (2 sites)	10/26/21-11/30/21 (4 sites)<br>12/16/21-2/7/22 (4 sites)<br>5/5/22-6/24/22 (2 sites)<br>6/8/22-7/29/22 (2 sites)
3	10/25/21-12/5/21 (6 sites)<br>11/19/21-12/27/21 (2 sites)<br>12/17/21-1/25/22 (2 sites)	6/8/22-7/29/22 (6 sites)<br>5/17/22-7/8/22 (2 sites)<br>6/14/22-7/29/22 (2 sites)

© 2024 Meredith LS et al. *JAMA Network Open*

eTable 2. Unadjusted Outcomes Pre- and Post-Intervention by Treatment Status for HCWs in FQHCs[a,b]

Outcome Measures (mean/SD)	Pre-Intervention			Post-Intervention		
	UC (N=183)	SFA (N=245)	p-value	UC (N=183)	SFA N=245)	p-value
**Primary Outcomes**						
Psychological distress score (range: 0-24)	5.929/6.395	6.620/6.715	0.284	6.306/6.799	6.469/6.750	0.806
Serious psychological distress (cutoff: >13)	14.8%	16.5%	0.619	17.5%	18.9%	0.703
PTSD symptom score (0-80)	12.425/14.937	15.456/16.591	0.055	12.836/15.251	14.792/16.904	0.224
PTSD provisional diagnosis (cutoff: >34)	9.5%	16.9%	0.030	10.7%	15.8%	0.134
PTSD (met DSM-5 clinical criteria)	12.8%	19.0%	0.094	12.4%	17.9%	0.127
**Secondary Outcomes**						
Sleep-related impairment score (range: 4-20)	8.060/4.394	8.820/4.701	0.090	7.995/4.430	8.563/4.476	0.192
Workplace stress score (range: 4-20)	7.060/2.962	7.299/2.655	0.381	7.656/3.227	7.935/2.677	0.329
Burnout, %	39.3%	36.1%	0.488	39.9%	37.6%	0.623
Resilience (range: 0-8)	6.443/1.536	6.008/1.677	0.006	6.426/1.484	5.812/1.674	<.0001
Moral distress (range: 0-10)	2.601/2.787	2.831/2.771	0.399	2.749/2.913	3.201/2.985	0.118

[a]UC stands for usual care; SFA stands for stress first aid; DSM-5 stands for Diagnostic and Statistical Manual of Mental Disorders (Fifth Edition); FQHCs stands for Federally Qualified Health Center; HCWs stands for Health Care Workers; PTSD stands for posttraumatic stress disorder.
[b]p-values are for the differences between UC and SFA at each wave.

© 2024 Meredith LS et al. *JAMA Network Open*

eTable 3. Unadjusted Outcomes Pre- and Post-Intervention by Treatment Status for HCWs in Hospitals[a,b]

Outcome Measures (mean/SD)	Pre-Intervention			Post-Intervention		
	UC (N=1,032)	SFA (N=617)	p-value	UC (N=1,032)	SFA (N=617)	p-value
**Primary Outcomes**						
Psychological distress score (range: 0-24)	5.986/5.473	5.556/5.226	0.117	5.735/5.515	5.735/5.511	0.998
Serious psychological distress (cutoff: ≥13)	11.78%	10.24%	0.339	9.80%	10.59%	0.607
PTSD symptom score (range: 0-80)	17.099/16.586	16.36/15.870	0.378	15.782/16.167	15.555/15.999	0.783
PTSD provisional diagnosis (cutoff: ≥34)	17.37%	15.30%	0.276	15.79%	17.13%	0.479
PTSD (met DSM-5 clinical criteria)	18.66%	16.61%	0.298	16.09%	18.60%	0.193
**Secondary Outcomes**						
Sleep-related impairment score (range: 4-20)	9.653/4.533	9.833/4.606	0.438	9.563/4.501	9.531/4.600	0.891
Workplace stress score (range: 4-20)	7.780/2.900	7.432/2.791	0.017	7.98/2.829	7.791/2.730	0.184
Burnout, %	48.35%	46.92%	0.573	50.05%	48.78%	0.619
Resilience (range: 0-8)	6.274/1.443	6.388/1.347	0.113	6.216/1.437	6.313/1.386	0.180
Moral distress (range: 0-10)	3.352/2.846	2.815/2.624	<0.001	3.167/2.797	2.852/2.791	0.027

[a]UC stands for usual care; SFA stands for stress first aid; DSM-5 stands for Diagnostic and Statistical Manual of Mental Disorders (Fifth Edition); HCWs stands for Health Care Workers; PTSD stands for post-traumatic stress disorder.
[b]p-values are for the differences between UC and SFA at each wave.

© 2024 Meredith LS et al. *JAMA Network Open*

eTable 4. Intention-to-Treat Results by Age Group for the Full Sample and by Facility Type[a,b]

Outcome measure (range or cutoff)	FQHCs (n = 428)	Hospitals (n = 1649)	Full sample (N = 2077)
	Estimate (95% CI)	Estimate (95% CI)	Estimate (95% CI)
**Age ≤30 y**			
**Primary outcome**			
Psychological distress score (range: 0-24)	−4.552 (-8.067, -1.037)[c]	0.465 (-0.662, 1.591)	−0.533 (-1.666, 0.601)
Serious psychological distress (cutoff: ≥13)	−1.270 (-2.460, -0.079)[c]	0.127 (-0.695, 0.950)	−0.153 (-0.852, 0.547)
PTSD symptom score (range: 0-80)	−6.771 (-13.224, -0.318)[c]	0.810 (-2.322, 3.942)	−0.674 (-3.477, 2.130)
PTSD provisional diagnosis (cutoff: ≥34)	−1.824 (-3.231, -0.435)[c]	0.068 (-0.468, 0.604)	−0.304 (-0.814, 0.206)
PTSD (met *DSM-5* clinical criteria)	−1.102 (-2.444, 0.240)	0.275 (-0.288, 0.838)	0.004 (-0.520, 0.528)
**Secondary outcome**			
Sleep-related impairment score (range: 4-20)	−0.128 (-2.374, 2.117)	−0.482 (-1.407, 0.444)	−0.411 (-1.272, 0.451)
Workplace stress score (range: 4-20)	−0.835 (-1.956, 0.285)	0.249 (-0.259, 0.727)	0.031 (-0.431, 0.493)
Burnout, %	−0.464 (-1.410, 0.482)	0.215 (-0.228, 0.657)	0.078 (-0.324. 0.479)
Resilience score (range: 0-8)	0.347 (-0.584, 1.278)	0.147 (-0.126, 0.421)	0.188 (-0.098, 0.473)
Moral distress score (range: 0-10)	−0.271 (-1.613, 1.071)	0.172 (-0.410, 0.755)	0.083 (-0.452, 0.618)
**Age 31-50 y**			
**Primary outcome**			
Psychological distress score (range: 0-24)	0.396 (-1.552, 2.344)	0.640 (-0.105, 1.385)	0.592 (-0.119, 1.302)
Serious psychological distress (cutoff: ≥13)	0.572 (-0.241, 1.385)	0.448 (-0.048, 0.944)	0.473 (0.044, 0.902)[c]
PTSD symptom score (range: 0-80)	0.359 (-3.033, 3.752)	−0.165 (-2.018, 1.690)	−0.061 (-1.689, 1.570)

© 2024 Meredith LS et al. *JAMA Network Open*

eTable 4. Intention-to-Treat Results by Age Group for the Full Sample and by Facility Type[a,b]

Outcome measure (range or cutoff)	FQHCs (n = 428)	Hospitals (n = 1649)	Full sample (N = 2077)
	Estimate (95% CI)	Estimate (95% CI)	Estimate (95% CI)
PTSD provisional diagnosis (cutoff: ≥34)	0.210 (-0.621, 1.042)	0.303 (-0.076, 0.683)	0.285 (-0.061, 0.631)
PTSD (met *DSM-5* clinical criteria)	0.436 (-0.319, 1.191)	0.245 (-0.130, 0.620)	0.283 (-0.05, 0.620)
**Secondary outcome**			
Sleep-related impairment score (range: 4-20)	−0.293 (-1.403, 0.817)	−0.072 (-0.650, 0.506)	−0.116 (-0.628, 0.396)
Workplace stress score (range: 4-20)	0.024 (-0.579, 0.626)	0.185 (-0.151, 0.521)	0.153 (-0.141, 0.447)
Burnout, %	0.200 (-0.314, 0.715)	0.003 (-0.253, 0.259)	0.043 (-0.187, 0.272)
Resilience score (range: 0-8)	−0.412 (-0.810, -0.013)[c]	−0.015 (-0.194, 0.164)	−0.095 (-0.258, 0.069)
Moral distress score (range: 0-10)	0.389 (-0.382, 1.159)	0.302 (-0.066, 0.670)	0.319 (-0.012, 0.651)
**Age ≥51 y**			
**Primary outcome**			
Psychological distress score (range: 0-24)	1.265 (-1.576, 4.106)	−0.338 (-1.865, 1.190)	0.032 (-1.305, 1.369)
Serious psychological distress (cutoff: ≥13)	−0.281 (-2.123, 1.561)	−0.233 (-1.154, 0.689)	−0.244 (-1.070, 0.583)
PTSD symptom score (range: 0-80)	1.128 (-3.419, 5.675)	2.031 (-1.336, 5.398)	1.821 (-0.955, 4.597)
PTSD provisional diagnosis (cutoff: ≥34)	1.533 (-1.246, 4.312)	0.383 (-0.419, 1.185)	0.651 (-0.242, 1.543)
PTSD (met *DSM-5* clinical criteria)	0.673 (-2.181, 3.527)	0.649 (-0.108, 1.406)	0.654 (-0.228, 1.537)
**Secondary outcome**			
Sleep-related impairment score (range: 4-20)	−0.464 (-1.935, 1.008)	−0.364 (-1.302, 0.574)	−0.387 (-1.180, 0.406)
Workplace stress score (range: 4-20)	0.302 (-0.626, 1.230)	−0.042 (-0.574, 0.490)	0.037 (-0.422, 0.496)

© 2024 Meredith LS et al. *JAMA Network Open*

eTable 4. Intention-to-Treat Results by Age Group for the Full Sample and by Facility Type[a,b]

Outcome measure (range or cutoff)	FQHCs (n = 428)	Hospitals (n = 1649)	Full sample (N = 2077)
	Estimate (95% CI)	Estimate (95% CI)	Estimate (95% CI)
Burnout, %	−0.031 (-0.882, 0.820)	−0.268 (-0.766, 0.230)	−0.214 (-0.644, 0.216)
Resilience score (range: 0-8)	0.035 (-0.593, 0.609)	−0.232 (-0.598, 0.134)	−0.171 (-0.480, 0.139)
Moral distress score (range: 0-10)	−0.059 (-1.262, 1.143)	0.034 (-0.666, 0.733)	0.012 (-0.589, 0.614)

Abbreviations: *DSM-5, Diagnostic and Statistical Manual of Mental Disorders* (Fifth Edition); FQHC, Federally Qualified Health Center; PTSD, posttraumatic stress disorder.

[a]Estimate refers to the estimated coefficient for the interaction term between treatment status and time. The difference-in-differences effect is on a linear scale for continuous variables and is a log odds ratio for binary variables. Models adjusted for study design features, demographics, and professional characteristics.

[b]Please note that the estimates for serious distress for ages 31-50 years (overall) and for resilience in ages 31-50 years (in FQHCs only) are statistically significant; however, the magnitude of the effect is not clinically meaningful and the pattern is inconsistent and not seen across the primary outcomes.

[c]$p<0.05$

© 2024 Meredith LS et al. *JAMA Network Open*
