# Supplementary material for: Testing an Intervention to Improve Health Care Worker Well-Being During the COVID-19 Pandemic: A Cluster Randomized Clinical Trial
Source: JAMA Netw Open. 2024 Apr 30;7(4):e244192. doi: 10.1001/jamanetworkopen.2024.4192 (PMC11061774; doi:10.1001/jamanetworkopen.2024.4192)
Supplement: Supplement 3. — Data Sharing Statement [file jamanetwopen-e244192-s003.pdf]

Data Sharing Statement

Meredith. Stress Intervention and Health Care Worker Well-Being During the COVID-19 Pandemic. *JAMA Netw Open*. Published April 04, 2024.
doi:10.1001/jamanetworkopen.2024.4192

Data
Data available: Yes
Data types: Deidentified participant data, Data dictionary
How to access data: URL not available yet. Data will be deposited to the funder's archiving system at the University of Michigan.
When available: With publication

Supporting Documents
Document types: None

Additional Information
Who can access the data: Anyone requesting the data
Types of analyses: For any purpose.
Mechanisms of data availability: Through the Curation Team at University of Michigan
